# Aminopeptidase N/CD13 Crosslinking Promotes the Activation and Membrane Expression of Integrin CD11b/CD18 †

**DOI:** 10.3390/biom13101488

**Published:** 2023-10-06

**Authors:** Laura Díaz-Alvarez, Mariana Esther Martínez-Sánchez, Eleanor Gray, Erandi Pérez-Figueroa, Enrique Ortega

**Affiliations:** 1 Instituto de Investigaciones Biomédicas, Departamento de Inmunología, Universidad Nacional Autónoma de México, Mexico City 04510, Mexico; 2 Posgrado en Ciencias Biológicas, Unidad de Posgrado, Edificio D, 1° Piso, Circuito de Posgrados, Ciudad Universitaria, Mexico City 04510, Mexico; 3 Instituto Nacional de Enfermedades Respiratorias “Dr. Ismael Cosío Villegas”, Mexico City 14080, Mexico; 4 London Centre for Nanotechnology, Department of Physics and Astronomy, University College London, London WC2R 2LS, UK; 5 Laboratorio de Investigación en Inmunología y Proteómica, Hospital Infantil de México Federico Gómez, Mexico City 06720, Mexico

**Keywords:** CD13, CR3, CD11b, cell signaling, macrophages, bioinformatics, cytokines

## Abstract

The β2 integrin CD11b/CD18, also known as complement receptor 3 (CR3), and the moonlighting protein aminopeptidase N (CD13), are two myeloid immune receptors with overlapping activities: adhesion, migration, phagocytosis of opsonized particles, and respiratory burst induction. Given their common functions, shared physical location, and the fact that some receptors can activate a selection of integrins, we hypothesized that CD13 could induce CR3 activation through an inside-out signaling mechanism and possibly have an influence on its membrane expression. We revealed that crosslinking CD13 on the surface of human macrophages not only activates CR3 but also influences its membrane expression. Both phenomena are affected by inhibitors of Src, PLCγ, Syk, and actin polymerization. Additionally, after only 10 min at 37 °C, cells with crosslinked CD13 start secreting pro-inflammatory cytokines like interferons type 1 and 2, IL-12p70, and IL-17a. We integrated our data with a bioinformatic analysis to confirm the connection between these receptors and to suggest the signaling cascade linking them. Our findings expand the list of features of CD13 by adding the activation of a different receptor via inside-out signaling. This opens the possibility of studying the joint contribution of CD13 and CR3 in contexts where either receptor has a recognized role, such as the progression of some leukemias.

## 1. Introduction

CD13, or aminopeptidase N, is a cell membrane ectoenzyme that is considered a marker of the myelomonocytic lineage [1]. Most of its 960 aa are located extracellularly, roughly 25 aa constitute the transmembrane portion and only 7–10 aa correspond to the intracellular portion of the protein [2]. The intracellular and extracellular segments of CD13 have distinct functions. The enzymatic activity is located in the extracellular domains and accounts for CD13’s role in the processing of bioactive peptides. The intracellular portion, on the other hand, mediates signal transduction when the receptor is crosslinked. This signaling activity is independent of its peptidase activity. Thus, CD13 can mediate cellular processes like phagocytosis and the subsequent respiratory burst, cell migration, and adhesion [3,4,5]. Signal transduction takes place despite the shortness of the intracellular tail with only a single potential p-Tyr and the absence of classical signaling sequences like ITAMs. Due to its wide range of activities, CD13 is considered a “moonlighting” protein.

Complement receptor 3 (CR3, Mac-1, or integrin αM/β2) is a member of a group of heterodimeric membrane proteins called α/β integrins. It is composed of the peptides CD11b (exclusive to CR3) and CD18; thus, it is also called CD11b/CD18 [6]. CR3 is primarily expressed in leukocytes like neutrophils, monocytes, macrophages, and dendritic cells [7]. CR3 has two main physiological roles. First, it acts as a phagocytic receptor for particles and pathogens opsonized with iC3b complement fragments (reviewed in [8]). Second, it is an adhesion molecule that participates in leukocyte extravasation during inflammation due to its ability to bind ligands present in endothelial cells [9,10]. The activation of CR3, that is, the transition from its low-affinity to its high-affinity conformation, occurs either through ligand recognition (outside-in signaling) or via an intracellular signal coming from a different cell surface receptor (inside-out signaling). Some signaling molecules can participate in both CR3 inside-out and outside-in signaling, including Rap1, RIAM, Talin, Kindlin, and Syk [7,11,12,13,14].

The link between CD13 and CR3 is also supported by in vivo evidence, as both molecules can be found together in functional microdomains within the cell membrane called lipid rafts [15]. These structures are key to cell signaling since they bring components of specific pathways close together, thus decreasing the possibility of fortuitous activation or blocking of signals from other cascades (reviewed in [16,17]).

In summary: i) integrins like CR3 can be activated by the engagement of other receptors due to a mechanism known as inside-out signaling, as is the case for FcγRs [18,19], with which CD13 shares the function of primary phagocytic receptor as well as the activation of many signaling molecules, and ii) both CD13 and CR3 can mediate functions such as phagocytosis, adhesion, and respiratory burst. Moreover, CD13 and CR3 can be found in physical proximity as both are present in lipid rafts [15], which is a strong indicator of a functional relationship. Additionally, a few publications have shown a functional link between CD13 and integrins: Carrascal et al. demonstrated that the expression of CD13 is associated with that of integrin α_v_β_3_ in breast cancer [20], and Ghosh et al. [21] showed that CD13 modulates the trafficking of integrin β1 via IQGAP, ARF6, and EFA6 in Kaposi sarcoma and human cervical cancer epithelial cells. In this work, we report the existence of a previously undescribed signaling pathway that links CD13 and CR3 in human macrophages using an integrated analysis of bioinformatics and experimental data.

First, we ascertained that crosslinking CD13 using monoclonal antibodies causes the activation of CR3. Second, we established the existence of at least two levels of control for the activation of CR3 following CD13 crosslinking: one that involves the inside-out signaling cascade that directly links both receptors and the second one that regulates the membrane expression of CR3. Third, we measured a panel of 12 cytokines and showed that, even at a short time after CD13 stimulation, CR3 activation is accompanied by the secretion of pro-inflammatory cytokines. Fourth, we used the information yielded by experiments along with the interrogation of molecular ontology bioinformatic databases, text mining analyses, and a manually curated functional protein interaction network, to suggest the components of the signal transduction pathway that leads to the activation and membrane expression of CR3 following CD13 crosslinking. A summary of our workflow can be found in Supplementary Figure S1.

Our findings have implications for the study of conditions in which the expression of CD13 is related to disease progression, as it is in breast cancer, where CD13 is linked to the development of metastases [20], a phenomenon largely driven by integrins.

## 2. Materials and Methods

### 2.1. Reagents and Antibodies

The RPMI-1640 medium was purchased from Gibco Life Technologies (Carlsbad, CA, USA). Recombinant human (rh) M-CSF was from PeproTech (Cranbury, NJ, USA). Lymphoprep was from Axis-Shield PoC AS (Oslo, Norway). All culture media were supplemented with 10% heat-inactivated FBS (Invitrogen, Carlsbad, CA, USA) unless otherwise stated, 2 mM L-glutamine, 100 μg/mL streptomycin, 100 U/mL penicillin (Sigma-Aldrich, St. Louis, MO, USA), 1 mM sodium pyruvate solution, and 1% MEM non-essential amino acids solution (100X) (Gibco by Life Technologies, Grand Island, NY, USA). Murine monoclonal IgG1 anti-human CD13 (Mab C) and anti-human CD32 (Mab IV.3) were produced and purified in our laboratory from supernatants of the corresponding hybridomas [4]. Fab fragments were prepared from the purified antibodies with immobilized Ficin (Pierce, Rockford, IL, USA), following the manufacturer’s instructions. Murine monoclonal APC anti-human CD11b (IgG1, clone ICRF44) and FITC anti-human CD11b (activated) antibody (IgG1, clone CBRM1/5) were from Biolegend (San Diego, CA, USA). Goat anti-mouse (GaM) polyclonal IgG F(ab)’2 fragments were from Jackson ImmunoResearch (West Grove, PA, USA). Polyclonal FITC rabbit anti-mouse antibody was purchased from Thermo Fisher Scientific (Waltham, MA, USA). Src inhibitor-1, U-73122 hydrate, Cytochalasin D, and BAY 61-3606 were from Sigma-Aldrich (St. Louis, MO, USA).

### 2.2. Cell Culture

Tohoku Hospital Pediatrics-1 (THP-1) cells (ATCC) were maintained in a humidified atmosphere at 37 °C with 5% CO_2_ in RPMI-1640 medium complemented as recommended by the selling company. For differentiation into macrophages, cells were seeded at 4.5 × 10^6^/10 cm plate or 8 × 10^5^/well in 6-well plates, in complemented RPMI-1640 medium and stimulated with 20 nM phorbol 12-myristate 13-acetate (PMA) for three days. Cells were washed once with warm PBS and incubated with fresh medium for 24 h before use. Differentiation was confirmed with CD11b expression (Supplementary Figure S2). All experiments carried out with cells from human donors were performed following the Ethical Guidelines of the Instituto de Investigaciones Biomédicas, UNAM, Mexico City, Mexico. Human peripheral blood mononuclear cells (PBMCs) were isolated from anonymous healthy male donors’ buffy coats obtained from the blood bank at Instituto Nacional de Ciencias Médicas y Nutrición Salvador Zubirán, Secretaría de Salud, Mexico City, Mexico, using gradient centrifugation with Lymphoprep, as previously described [4]. For monocyte isolation, PBMCs were washed three times with PBS, pH 7.4, using centrifugation at 400× *g* for 10 min. After the last wash, cells were resuspended in serum-free RPMI-1640 medium complemented as described before and were seeded (5–6 × 10^7^ PBMCs/plate) in 100 mm × 20 mm cell culture-treated polystyrene culture dishes (Corning, New York, NY, USA). Cultures were maintained in a humidified atmosphere at 37 °C with 5% CO_2_ for 1 h to allow monocytes to adhere to the plastic plate. Non-adherent cells were eliminated with gentle washing, and adherent cells, enriched for monocytes (≥95% purity, as determined with flow cytometry using CD14 as a marker of the monocytic population. were cultured for 7–10 days, for differentiation into macrophages, in RPMI-1640 medium complemented as described before plus 5 ng/mL rh M-CSF at 37 °C. For experiments, macrophages were harvested with gentle cell scraping.

### 2.3. CR3 Activation

The cells were incubated for 3 h in serum-free supplemented RPMI-1640 with or without inhibitors (10 mM BAY, 20 mM Src inhibitor-1, 5 mM U-73122 hydrate, or 10 mM cytochalasin D). Then, they were harvested. Freshly harvested macrophages incubated without inhibitors were called “pre-treatment”. Thus, the treatment consisted of incubating 0.25 × 10^6^ cells/sample in 0.2 mL serum-free supplemented RPMI-1640 medium with 2.5 mg of mAb C (anti-CD13) or mAb IV.3 (anti-CD32, positive control) complete antibody for 30 min at 4 °C. The cells were washed three times with fresh medium and incubated with 4 mg of GaM F(ab)’2 fragments for 30 min at 4 °C. Immediately after, the cells were incubated for 10 min at 37 °C and then pelleted before being fixed with 1% paraformaldehyde (PFA) for 25 min at RT. The cell-free supernatants from certain samples were collected and stored at −20 °C for cytokine quantification (see below).

### 2.4. Flow Cytometry

To quantitate CD11b expression and activation, fixed samples were washed two times with cold PBS and stained with 50 mL of a 1:20 dilution of murine monoclonal APC anti-human CD11b (IgG1, ICRF44) or FITC anti-human CD11b (activated) antibody (IgG1, CBRM1/5) for 40 min at 4 °C. The cells were washed three times with cold PBS. Staining for CD13 or CD32 (FcgRII) was performed by incubation in 10 mg anti-CD13 or anti-CD32 mAbs in serum-free supplemented RPMI-1640 medium for 30 min at 4 °C. The cells were washed three times with the same medium, incubated with 1:500 GaM-FITC antibody for 30 min at 4 °C, and then washed three times with cold PBS and fixed with 1% PFA for 25 min at RT. Fluorescence intensity was measured using flow cytometry (Blue/red Attune cytometer, Applied Biosystems-Thermo Fisher, Waltham, MA, USA). Flow cytometry data were displayed either as MFI for cells with crosslinked CD13 vs. their activation controls or as integrated MFI (iMFI, percentage of positive cells multiplied by their MFI) for cells with CD13 crosslinked in the presence of inhibitors [22]. For the latter, a normalized proportion of the cells incubated with inhibitors vs. their respective controls is presented.

### 2.5. Cytokine Quantification

Samples from the CR3 activation experiments were used to quantify a panel of 12 cytokines. Specifically, supernatants from cells without antibodies (control) and cells with both primary and secondary antibodies (Mab C + sec) were assayed. The frozen supernatants were carefully thawed in ice and loaded as duplicates onto two Milliplex plates (Millipore Sigma, Darmstadt, Germany), one to detect IFN-a and a second one for IFN-g, IL-12p70, IL-17a, IL-6, IL-1b, IL-2, IL -8, IL-4, IL-10, MCP-1, and TNF-a. The assays were performed according to the manufacturer’s instructions and measured in a Luminex Multiplexing Instrument (Millipore Sigma, Darmstadt, Germany).

### 2.6. Theoretical Cell Signaling Interaction Network Assembly

We constructed the functional protein interaction network of CD13, Syk, and CR3 and their closest partners using combined interaction scores from STRING [23]. A functional association in this context means either physical contact, participation in the same metabolic pathway, and/or cellular process [24]. STRING scores are indicators of text mining and protein homology. Each type of evidence gives rise to an individual score for each likelihood of an interaction given currently available evidence in the database, which includes gene neighborhood, gene fusions, gene co-occurrence, experimental evidence, curated databases, and pairs of proteins. STRING computes combined scores by integrating the individual scores and correcting for the probability of randomly observing the interaction. Scores rank from 0 to 1, with 1 being the highest possible result.

The search for functional partners was performed individually for each interrogation query (CD13, CD11b, CD18, and Syk) and focused on human proteins. High confidence scoring molecules (0.8 and above) from the first layer of interactions with the query were considered. The resultant group of proteins was filtered based on the requirements for this particular inside-out signaling pathway: non-receptor kinases, adaptor proteins able to bridge CD13 to other components of the pathway, especially Syk, and inhibitory molecules like protein phosphatases or ubiquitin ligases. In some cases, other interacting receptors were considered, as they may provide insight into the reported mechanisms for this type of interaction. Namely, those similar to the studied receptors, CD13 and CR3: metalloproteases, phagocytic receptors, integrins, and other adhesion molecules. To ensure the quality and specificity of the network text mining STRING element, The GeneCards website [25] and the repository PubMed [26] were used to ascertain the suitability of each selected protein, i.e., to confirm the function of each node, as well as its gene and protein expression in myelomonocytic cells. Finally, the interaction network was manually curated according to experimental evidence gathered from previous publications.

### 2.7. Statistical Analysis

Statistical analyses were performed using one-way ANOVA followed by a multiple comparisons test or a paired two-tailed *t*-test in the case of experiments with MDMs and cytokines. *p* values below 0.05 were considered significant.

## 3. Results

### 3.1. Crosslinking CD13 Results in the Activation of CR3 (CD11b/CD18)

**Figure 1:**
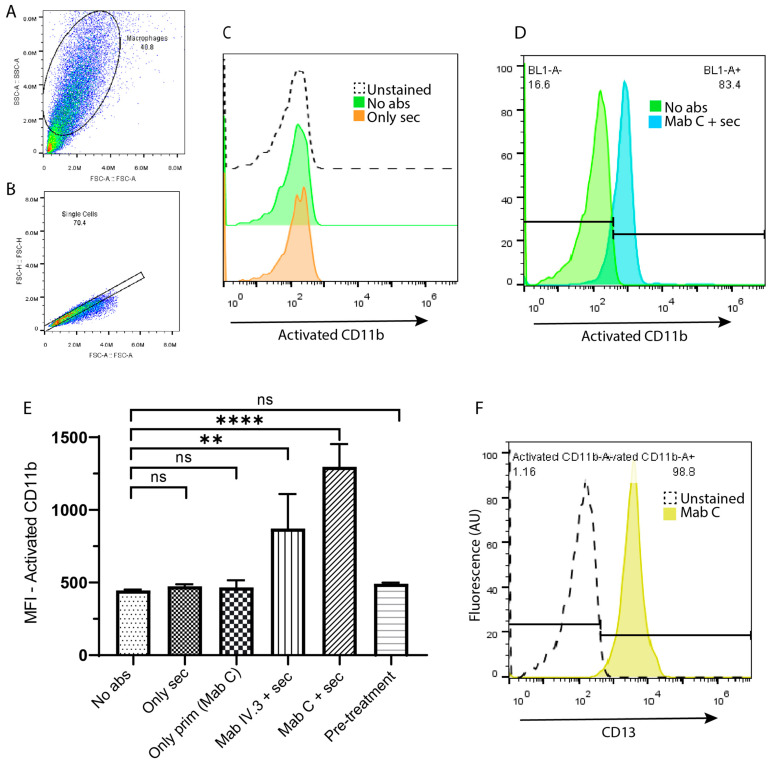
CD13 crosslinking activates CR3 in human THP-1 macrophages. (**A**) Cells were first gated for size and granularity, then for (**B**) singlets, and finally, for (**C**,**D**) MFI in the BL1 (FITC) channel. (**C**) Controls. (**D**) Representative histograms from a sample crosslinked with C (anti-CD13) and secondary antibodies vs. its control without antibodies. (**E**) Average and SDs from 3 independent experiments. ** *p* < 0.01, **** *p* < 0.0001, ns = non-significant. (**F**) Representative histogram demonstrating that virtually all cells are positive for the CD13 stain.

We assessed the activation status of CR3 (CD11b/CD18) following CD13 crosslinking on human macrophages. CD13 molecules on the surface of THP-1 macrophages were crosslinked using the complete anti-CD13 antibody mAb C as the primary antibody and GαM F(ab)’2 fragments as the secondary antibody. Next, the cells were stained with a FITC-anti-CD11b (activated) antibody and analyzed using the flow cytometer. The cells were first gated for size and granularity (Figure 1A), then for singlets (Figure 1B), and finally, for median fluorescence intensity in the BL1 (FITC) channel (Figure 1C,D). Figure 1C shows that the fluorescence histogram for the control unstimulated cells stained with anti-CD11b (activated) antibody overlaps with the auto-fluorescence of unstained cells. The controls are cells incubated either without antibodies or only with secondary antibodies. The resulting histograms demonstrate that incubation in the absence of an anti-CD13 antibody does not produce a nonspecific anti-CD11b (activated) signal. In contrast, panel D shows a representative histogram for the CR3 activation produced when CD13 is crosslinked using both primary and secondary antibodies. Figure 1E shows the average and SD in the MFI from CR3 activation in CD13-crosslinked cells (*n* = 3) along with its controls. A one-way ANOVA followed by a multiple comparisons test confirms that our negative controls, i.e., cells incubated with only primary or secondary antibodies, as well as freshly harvested macrophages (“pre-treatment”), show no significant difference with cells incubated without antibodies. Only the activation of CR3 in cells with either crosslinked CD32 (positive control [18,19]) or CD13 is significantly different from that in cells without antibodies (<0.01 and <0.0001, respectively). Figure 1F is a representative histogram for THP-1 macrophages incubated with Mab C and a secondary antibody coupled to FITC, showing that Mab C bounds efficiently to all cells. These results were consistent in MDMs (Supplementary Figure S3).

### 3.2. Syk, Src, PLCγ, and Actin Polymerization Participate in the Activation of CR3 Triggered by CD13 Crosslinking

In order to gain insight into the signaling pathway connecting CD13 crosslinking and the activation of CR3, we chemically inhibited some of the molecules related to the signaling of these receptors.

**Figure 2:**
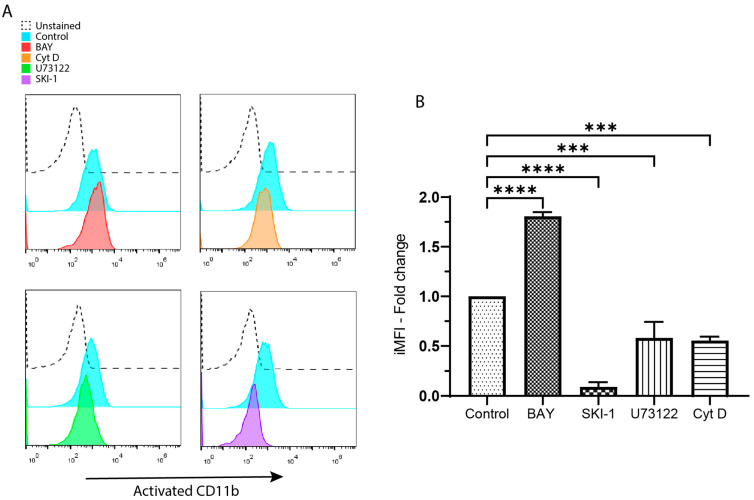
The inhibition of Src, PLCγ, and actin polymerization reduces the activation of CR3 (CD11b/CD18) triggered by CD13 crosslinking. The inhibition of Syk augments it. (**A**) Representative histograms from the activation of CR3 on cells with CD13 crosslinked in the presence of inhibitors for Syk (BAY), actin polymerization (Cyt D), PLCγ (U73122), and Src (SKI-1). (**B**) Average and SDs for the iMFI from 3 independent experiments. *** *p* < 0.001, **** *p* < 0.0001.

We assessed how these inhibitors affect the activation of CR3 triggered by CD13 crosslinking. For this, we pre-incubated THP-1 macrophages with either BAY 61-3606 (BAY), Src kinase inhibitor-1 (SKI-1), U73122, cytochalasin D (Cyt D), or no inhibitor (control) for 3 h in a serum-free medium. Then, the cells were harvested and CD13 on their surface was crosslinked. Finally, we measured CR3 activation using flow cytometry. Figure 2A shows representative histograms comparing cells stained with the anti-CD11b (activated) with or without inhibitors. BAY augments the signal, while Cyt D, SKI-1, and U73122 diminish it. Such differences were statistically confirmed and are represented in Figure 2B, where the average and SD of the proportion of each inhibitor-incubated sample vs. their respective control is plotted. The *p*-values for both BAY and SKI-1 are <0.0001 and <0.001 for both Cyt D and U73122 (*n* = 3). These results indicate that Syk, Src, PLCγ, and actin polymerization have a role in the activation of CR3 triggered by CD13 crosslinking. It is noteworthy that incubation with BAY had the same effects on human MDMs (Supplementary Figure S4).

### 3.3. CD13 Crosslinking Also Controls CR3 Membrane Expression

**Figure 3:**
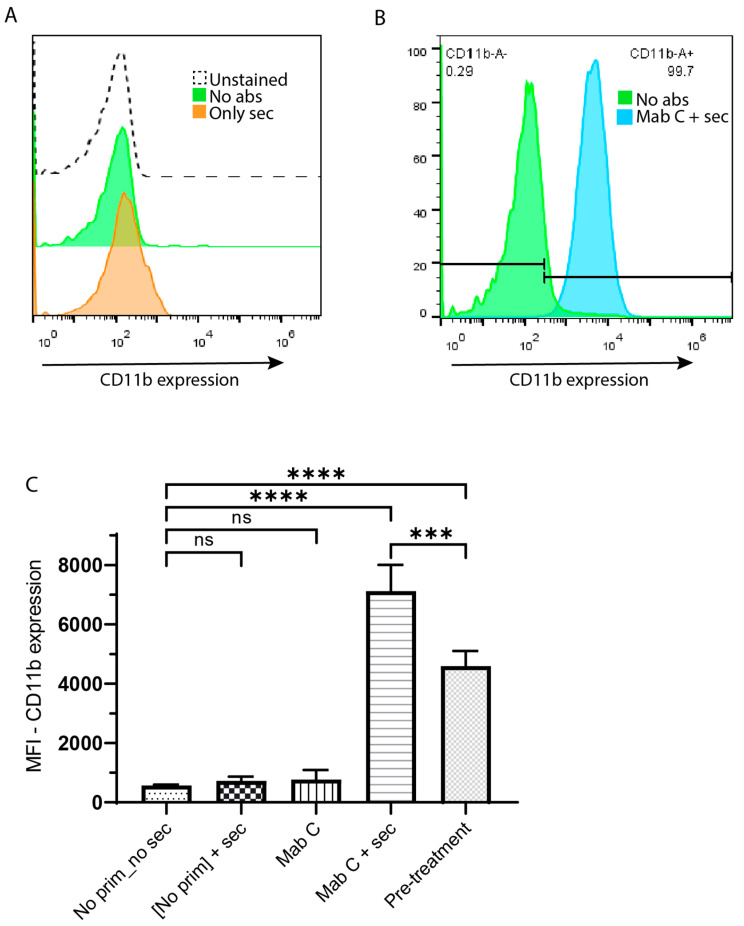
CD13 crosslinking promotes CR3 membrane expression in THP-1 macrophages. (**A**) MFI in the RL1 channel (APC) from unstained cells and, control cells treated without crosslinking antibodies or only with secondary antibody, stained with anti-CD11b(total). (**B**) Representative histograms from a sample crosslinked with mAb C (anti-CD13) and secondary antibodies vs its control without antibodies. (**C**) Average ±SDs of MFIs of CD11b (total) expression on cells treated as indicated in the graph or non-treated cells. Data from 3 independent experiments. *** *p* < 0.001, **** *p* < 0.0001. ns = non-significant.

In order to determine if CD13 crosslinking had any effect on the membrane expression of CR3, we evaluated CR3 membrane expression using flow cytometry in THP-1 macrophages. Figure 3A displays representative histograms showing that the signal from the controls (cells incubated without antibodies or only with secondary antibody) stained with an a-CD11b antibody coupled to APC practically overlaps with that of unstained cells. In contrast, Figure 3B shows that crosslinking CD13 on the surface of macrophages induces the surface expression of CR3. Figure 3C shows the average and SD for the MFIs of CD11b expression on freshly harvested macrophages (pre-treatment), control cells, and cells in which CD13 was crosslinked from three independent experiments. The overall expression of CR3 exhibits the same pattern as CR3 activation, except for cells before treatment. Cells stained before treatment have a basal CR3 level significantly different from that of cells treated without antibodies (control). These data indicate that basal CD11b membrane expression decreases after treating cells (two 30′ incubations at 4 °C, three washes, and a 10′ incubation at 37 °C) in the absence of antibodies or only with primary or secondary antibodies. Only CD13 crosslinking restores CR3 membrane expression, even at a higher level than pre-treatment.

### 3.4. Src, PLCγ, Syk, and Actin Polymerization Also Have a Role in CR3 Membrane Expression

**Figure 4:**
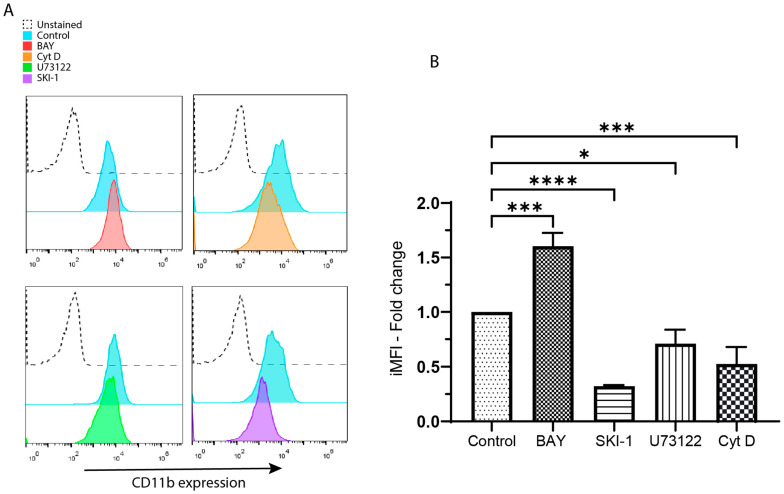
The inhibition of Src, PLCγ, and actin polymerization reduces the membrane expression of CR3 (CD11b/CD18) triggered by CD13 crosslinking. The inhibition of Syk augments it. (**A**) Representative histograms from the membrane expression of CR3 on cells with CD13 crosslinked in the presence of inhibitors for Syk (BAY), actin polymerization (Cyt D), PLCγ (U73122), and Src (SKI-1). (**B**) Average and SDs of the iMFI from 3 independent experiments. * *p* < 0.0221, *** *p* < 0.001, **** *p* < 0.0001.

After confirming that CD13 crosslinking also influences CR3 membrane expression, we investigated the possibility that the signaling pathway controlling this phenomenon and the one governing the activation of CR3 shared some of their components. For this, we pre-incubated THP-1 macrophages with either BAY, SKI-1, U73122, Cyt D, or no inhibitor (control) for 3 h in a serum-free medium. Then, the cells were harvested and CD13 on their surface was crosslinked. Finally, we measured CR3 membrane expression using flow cytometry. Figure 4A shows representative histograms comparing cells stained with anti-CD11b (total) after stimulation by CD13 crosslinking in the presence of the different inhibitors. The pattern is similar to the one observed for CR3 activation: BAY augments the signal produced by the fluorochrome-coupled antibody in comparison with the control, while Cyt D, SKI-1, and U73122 diminish it. Such differences were statistically confirmed and are represented in Figure 2B, where the average and SD for the proportion of each inhibitor-incubated sample vs. their respective control is plotted. The *p*-value for SKI-1 was <0.0001, <0.001 for both BAY and Cyt D and, 0.0221 for U73122 (*n* = 3). These results indicate that Syk, Src, PLCγ, and actin polymerization have a role in CR3 membrane expression influenced by CD13 crosslinking. Supplementary Figure S5 shows a comparison of the relation CD11b activation/expression in cells crosslinked in the presence of the different inhibitors.

### 3.5. CR3 Activation Triggered by CD13 Crosslinking Is Accompanied by the Secretion of Inflammatory Cytokines

**Figure 5:**
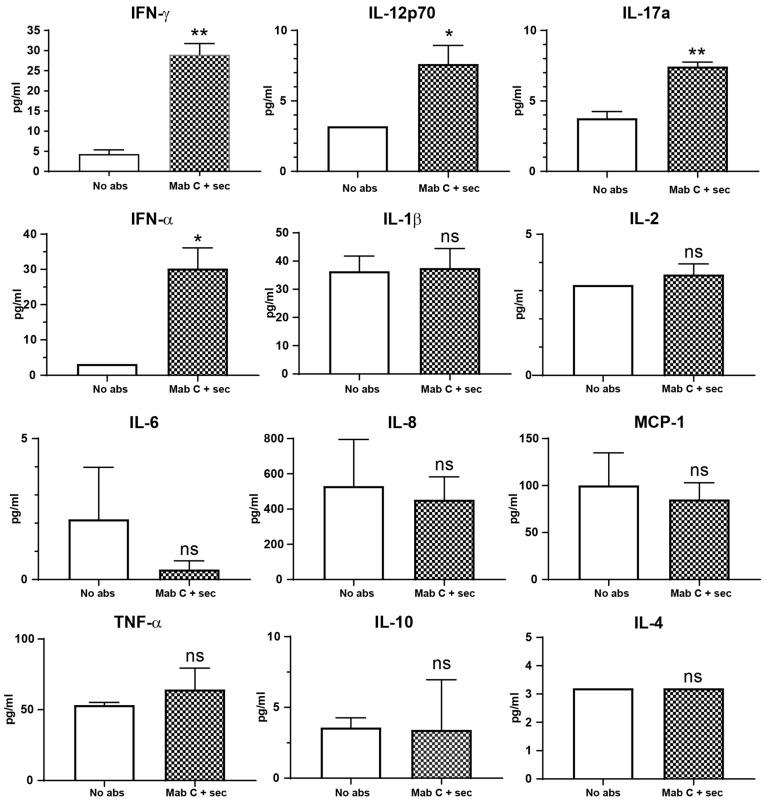
The activation of CR3 (CD11b/CD18) triggered by CD13 crosslinking is accompanied by the secretion of pro-inflammatory cytokines. Quantification of 12 cytokines present in the cell-free supernatant of cells with CD13 crosslinked and their control cells treated without antibodies. * *p* < 0.05 (0.0154 for IFN-α, and 0.0283 for IL-12p70), ** *p* < 0.01, ns = non significant. Average and SDs from 3 independent experiments.

Immune cells commonly respond to stimuli by secreting cytokines. The array of secreted cytokines determines the events that will follow the original stimulus (e.g., pro-inflammatory or anti-inflammatory). This is the reason why these proteins largely help orchestrate the local and systemic response. Thus, it is of interest to know the milieu generated, i.e., the accompanying cytokine profile, when immune receptors activate, in this case, CR3. This does not mean that the activation and rise in membrane expression of CR3 triggered by CD13 crosslinking are driven by cytokine secretion, rather, they are part of the overall cell response to a single stimulus. To this effect, we measured a panel of 12 cytokines. The cell-free supernatants of cells incubated without antibodies (control) and incubated with primary and secondary antibodies (Mab C + sec) were used to determine IFN-α, IFN-γ, IL-12p70, IL-17a, IL-6, IL-1β, IL-2, IL -8, IL-4, IL-10, MCP-1, and TNF-α. Only the pro-inflammatory cytokines IFN-α (*p* = 0.0154), IFN-γ (*p* < 0.01), IL-12p70 (*p* = 0.0283), and IL-17a (<0.01) had a significant increase in their concentration compared with the control, as seen in Figure 5. IFNs reached an average of 30 pg/mL, and IL-12p70 and IL-17 reached an average of 8 pg/mL. Even though other cytokines like IL-8, TNF-α, and MCP-1 have higher concentrations, these were not significantly different from their controls.

### 3.6. The Interaction Network for CD13, Syk, and CR3 (CD11b/CD18) Functional Partners Contains 76 Proteins

The previous results showed that crosslinking CD13 on human macrophages induced the high-affinity conformation of CR3 and its membrane expression. Thus, we turned to bioinformatic databases to assemble an interaction network composed of functional partners of CD13, CR3, and Syk, one of the molecules explored in our chemical inhibition assays and a key signaling kinase in the immune system, particularly in myeloid cells, to propose a sequential mechanistic model for the inside-out signaling pathway that could account for the activation of CR3 following CD13 crosslinking.

To determine the potential set of proteins and pathways that participate in the CD13-CR3 inside-out-signaling cascade, we constructed an interaction network using information from public databases, the literature, and previous experimental work from our laboratory. Given the high number of potential candidates, network nodes were selected using the predicted interaction score, biological function, and presence in the target cell type.

A functional protein interaction network for CD13, Syk, and CR3 was assembled by selecting the proteins with the highest combined scores (0.8 or more) from the STRING database [23], as well as previously determined experimental interactions. Data mining the STRING element and the databases GeneCards [25] and PubMed [26] were used to confirm that the chosen proteins were present in the myelomonocytic lineage. Supplementary Figure S6 presents the main ontology clusters for the selected proteins. For those interrogation nodes that resulted in more than 50 proteins with combined scores ≥0.8, the top 50 molecules were analyzed.

Using Syk as the interrogation query, we obtained a first layer of interactions among 158 proteins and STRING combined scores above 0.9. Twenty-nine entries were selected according to the established criteria, i.e., representing CD13 and/or CR3 known functional interactors or potential elements for the inside-out signaling pathway connecting the two of them. Two of these proteins were also selected in the CD11b and CD18 analyses. Supplementary Figure S7 includes the proteins selected to assemble the network, and the Venn diagram allows the identification of those molecules common to two or more interrogation queries. In the case of Syk, two of its interactors were also common with CD11b and CD18.

Polypeptide chains forming CR3 (CD11b (ITGAM) and CD18 (ITGB2)), were also subjected to this type of analysis. For CD11b, the first layer of interactions with STRING combined scores above 0.9 consisted of 167 proteins, resulting in 22 molecules of interest. Ten of these were also selected for CD18, as well as the two previously mentioned for both Syk and CD18.

Using CD18 as the interrogation query resulted in 184 interactors with a combined score of ≥0.9. Twenty-five molecules of interest were chosen, 13 of which were exclusive to CD18, and the rest were shared with Syk and CD11b, as aforementioned.

Using CD13 as the interrogation query yielded 27 molecules with STRING combined interaction scores of 0.8 and above; these were filtered to four proteins of interest using the criteria of being either downstream signal inhibitors or enhancers, adhesion molecules, or co-receptors which, following text mining, might provide information on the signaling pathways necessary for interaction with CD13. Finally, 12 proteins for which an interaction with CD13 was previously experimentally determined (SYK, GRB2, PI3K, FAK, IQGAP1, SRC, JNK, p38, MEK-1, PKC, ERK ½, and SOS1) were added to the molecules of interest [5,27,28].

**Figure 6:**
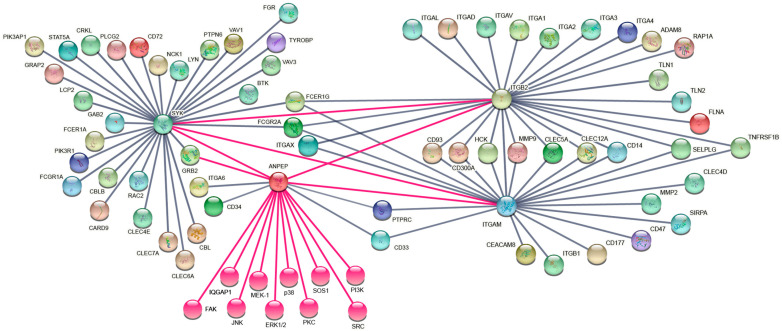
The interaction network for CD13, Syk, and CR3 functional partners contains 76 proteins. Nonredundant results from the analysis of the STRING highest scoring interacting partners among CD13, Syk, and CR3. Pink lines and bubbles represent the interactions experimentally determined in our laboratory and by others.

Figure 6 depicts the interaction network obtained, consisting of 76 non-redundant proteins. Of note, pink lines and bubbles represent interactions experimentally determined, including the ones contributed by this study. A detailed list of all proteins in the network, their main characteristics, and their corresponding interrogation nodes, is presented in Table S1.

Next, based on our interaction network, we constructed a sequential mechanistic model of the CD13-CR3 inside-out signaling pathway (Supplementary Figure S8).

## 4. Discussion

CD13 is an ectopeptidase that, along with other proteins like CD157, CD73, CD38, and CD26, can initiate signaling events upon stimulation [27,29]. Despite the need for extra accessory proteins, the existence of receptors without tyrosine-kinase activity (non-RTKs) like these ectopeptidases, may have been retained during evolution as they provide a tighter cell activation control than receptor tyrosine kinases (RTKs). Unlike non-RTKs, RTKs can undergo spontaneous activation upon stochastic encounters in the cell membrane, which poses a risk when they are overexpressed, as they can lead to disease development. For instance, overexpression of the RTK human epidermal growth factor receptor 2 (HER2) is associated with various cancers, including ovarian, prostatic, gastric, lung, and breast cancers. Furthermore, HER2 activation serves as a known mechanism of resistance to endocrine treatment in experimental models [30].

CR3 can exist in two main conformational states that correspond to a high or low affinity for its ligands, referred to as the active and inactive states, respectively. The high-affinity state can be reached either by outside-in or inside-out signaling (reviewed in [31,32]). We hypothesized that CD13 could functionally interact with integrins like CR3 by promoting its activation, considering the following two facts. First, the stimulation of many immune receptors activates CR3 via inside-out signaling, including but not limited to CD14, TLR2, TLR4, TLR9, and FcγRs [19,33,34]. Second, CD13 functionally interacts with other immune receptors, for example, crosslinking CD13 with monoclonal antibodies increases the phagocytic efficiency of particles directed to FcγRs [35]. Of note, due to the lack of reported natural ligands that stimulate CD13 signal transduction, so far crosslinking has been the stimulus of choice for this receptor [5,27,36].

We found that CD13, a non-RTK with a short cytoplasmic tail and no canonical signaling motifs [28], activates CR3 and controls its membrane expression. CD11b membrane expression does not always indicate activation, as shown by the different CD11b activation/total CD11b ratios in cells treated with various inhibitors. This supports the idea that CD13 crosslinking triggers two separate phenomena: expression involving Syk and actin polymerization and activation involving PLCγ. Notably, CD13 crosslinking is necessary to initiate these signaling events. In the absence of antibodies or with only primary or secondary antibodies, CR3 is most likely internalized and only recycled back to the membrane upon CD13 crosslinking, potentially through a clathrin-mediated mechanism.

CD11b levels increase when monocytes differentiate into macrophages, i.e., it is a differentiation marker. This partially explains the enhanced potential of macrophages for mobility, adhesion, and phagocytosis, compared to their precursors [6]. Upon stimulation of immune receptors, associated factors activate, and, in many cases, their gene expression increases [37]. However, crosslinking CD13 on the surface of macrophages at 4 °C, followed by a brief ten-minute incubation at 37 °C, results in an even higher expression of CD11b on the cell membrane than the baseline differentiation levels (referred to as “pre-treatment” in our experiments). The interpretation of this phenomenon is that, first, certain in vivo scenarios require that CD11b membrane levels increase at shorter times than those allowed by gene expression. Thus, the existence of receptor reservoirs in the form of intracellular vesicles [38]. Second, CD13 may function as a sentinel, detecting stimuli that require the activation and involvement of CD11b, thereby facilitating a swift response to immunological challenges.

Subramani et al. [5] demonstrated that crosslinking CD13 on the human monocytic cell line U937 induces adhesion to endothelial cells and that this phenomenon is related to the phosphorylation of the receptor by Src, as well as to the recruitment of cytoskeleton-binding machinery. Thus, the selection of SKI-1 and Cyt D. BAY, a highly selective and widely used Syk inhibitor [39,40,41], was tried because Syk acts downstream of several immune receptors on myelomonocytic cells, including CD13 and integrins [3,41]. In fact, Zheng et al. [41] showed that after the glycoprotein VI on human platelets engages its ligand collagen, an inside-out signaling pathway sets off, activating Syk, which phosphorylates PLCγ, leading to the activation of integrin a_IIb_b_3_. Thus, our choice to also include the PLCγ inhibitor U73122.

Our sequential mechanistic model is supported by both STRING-predicted interactions and previous experimental data, making the pathway theoretically conceivable. For example, we chose Grb2 as an adaptor molecule bridging CD13 and Syk based on our previous findings of crosslinked CD13 co-precipitating with Grb2. Also, this molecule associates with Shc, Src, Syk, and SHP-1 during inside-out signaling between CD32a and αIIbβ3 integrin in human platelets [42,43]. Similar pathways have been observed in other systems, such as human neutrophil PSGL-1 binding endothelial P- and E-selectins, thus activating β2 integrins CR3 and LFA-1 (reviewed in [7]). Another example is Rap1, chosen for being a key regulator of inside-out activation in phagocytic integrins like CR3, where signals from various receptors converge [44]. These findings highlight the effectiveness of combining experimental and bioinformatic approaches to unravel complex signaling pathways. Our model expands the understanding of the intricate inside-out signaling cascade.

Previous publications have linked cytokine production to the expression of CD13 in various cell types and contexts [45,46,47,48,49]. However, only a handful of studies have reported the secretion of cytokines as a result of CD13 stimulation in human myeloid cells. In a recent work from our group, Perez-Figueroa et al. [36] incubated human neutrophils for 24 h with the same primary and secondary antibodies we used in this study. A bead-based multiplex assay was used to determine the production of IL-1β, TNF-α, IL-8, IL-6, and IL-10 in cell-free supernatants. From these, only IL-1β and TNF-α showed a significant increase compared with the control. Similarly, Santos et al. [27] showed that the ligation of CD13 on U937 human monocytes upregulates the mRNA expression of IL-8, peaking at 2 h of incubation. In contrast, Villaseñor-Cardoso et al. [50] surveyed supernatants from human monocyte-derived DCs and macrophages for IL-6, IL-12, IL-10, and TNF-α, but found no increase in their concentration after 18 h of CD13 crosslinking. This result could be due to the use of sandwich ELISA, a less sensitive method. In this work, we measured a panel of 12 cytokines and detected the presence of pro-inflammatory cytokines accompanying the activation and rise in membrane expression of CR3 after only 10 min following CD13 crosslinking. Specifically, IFNs type 1 (IFN-α) and 2 (IFN-γ), IL-12p70, and IL-17a increased significantly. As expected for such a short time after stimulation, the concentrations were lower than what other authors have reported for THP-1 macrophages. For example, we detected approximately 8 pg/mL IL-12, whereas Shabir et al. [51] and Souissi et al. [52] reported that THP-1 macrophages produce 100–125 pg/mL IL-12, albeit after 4–18 h of stimulation with 100 ng/mL LPS, a potent pro-inflammatory cytokine inducer. Similarly, Zhou et al. [53] reported that 24 h after infection with Mycobacterium tuberculosis, THP-1 macrophages secret almost 300 pg/mL IL-12. Therefore, when both the time after stimulation and the method of detection are considered, it becomes evident that there is still a knowledge gap regarding the early cytokine response after CD13 crosslinking. Thus, time-course experiments and monitoring cytokine expression and secretion between 10 min and 18–24 h are necessary to determine if CD13 crosslinking can induce similar cytokine concentrations as other pro-inflammatory stimuli. Nevertheless, the cytokines we detected are biologically relevant to CD13-associated processes. Type 1 IFNs are canonical cytokines secreted as part of the antiviral response [54]; thus, it is expected that a viral receptor, like CD13 [55,56], drives its production. This is evidenced by the work of Yamaya et al. [57], who demonstrated that Type 1 IFN is secreted after the human coronavirus 229E, one of the many that cause common colds, engages its receptor CD13 on the surface of primary human nasal and tracheal epithelial cells. IL-12 is an early cytokine secreted by myeloid cells in response to PAMPs and DAMPs and induces the expression and secretion of IFN-γ (reviewed in [58,59,60]). Of note, the p70 subunit is also one of the two monomers constituting IL-23, another member of the IL-12 family of heterodimeric cytokines. In any case, both IL-12 and IL-23 contribute to the functions of Th1 and Th17 subsets of T lymphocytes, respectively. The secretion of these cytokines may also be related to CD13-induced endosome recycling, as these compartments are involved in the secretion of cytokines like TNF-α, IL-6, and IL-10 [61,62,63]. Nevertheless, the most intriguing of the CD13 crosslinking-induced cytokines is IL-17a. This is because it is primarily associated with the Th17 subpopulation of CD4+ T cells, where it was first described (reviewed in [64]). However, an increasing body of evidence indicates that macrophages and other myeloid cells express IL-17a [65,66,67]. Considering the pathophysiological significance of both IL-17a and macrophage recruitment in various conditions such as endometriosis, sepsis, and lung cancer [65,68,69], future research is warranted on the contribution of macrophage-derived-IL-17a in these contexts. Additionally, both IL-17 and IFN-γ are known to drive CD13 upregulation [49,70,71]. Of note, TNF-α, IL-8, and MCP-1 were detected at concentrations ranging from 50 to 500 pg/mL, but they did not increase significantly after crosslinking CD13. Thus, it is highly likely that the secretion of these cytokines is the result of the conditions to which the cells are subjected during the incubations, namely, changes in temperature and mechanical stress.

Our findings suggest that CD13 and CR3 (CD11b/CD18) may collaborate in various cellular functions, including adhesion. CD13 has long been implicated in pro-adhesive events such as aggregation [72,73] and invasiveness [74], while CR3 (CD11b/CD18) is well-known for its adhesive properties and activation in response to the stimulation of other receptors. For example, CD11b activation triggered by human neutrophil antigen 3a auto-antibodies leads to neutrophil accumulation in the pulmonary microvasculature of some blood transfusion recipients, causing severe transfusion-related acute lung injury [75]. This suggests that CD13 and CR3 may participate in the same adhesion events during inflammation-related transendothelial migration. Although our group previously reported that CD13-mediated adhesion to endothelial cells is integrin-independent [28], it is important to note that CD11b was not among the integrins evaluated. This could partially explain the observation that CD13 ligation impairs transendothelial migration in vivo [28]. Given that, as we demonstrated, CD11b activation is a consequence of CD13 stimulation, persistent CD13 engagement would render active CR3 in constant contact with its endothelial ligands such as ICAM-1 and ICAM-2, JAM-A, JAM-C, and RAGE [9]. This continuous engagement could lead to cell arrest, polarization, and spreading, potentially inhibiting extravasation [76]. While it remains uncertain, our findings suggest that CD11b may indeed contribute to this process. Further investigation is needed to confirm or rule out its involvement.

CD13 and CR3 may also collaborate in phagocytosis, as both receptors perform this cellular function. Licona-Limón and colleagues [3] demonstrated that CD13 is a primary phagocytic receptor, capable of mediating phagocytosis of CD13-directed phagocytic preys by human macrophages and THP-1 monocytes. Even when expressed in non-phagocytic HEK293 cells, CD13 enables them to internalize the same type of phagocytic particles. As for CR3, its involvement in the complement cascade is well-established. Activation of the complement system leads to the generation of opsonizing molecules like iC3b, which are recognized by CR3 to facilitate phagocytosis (reviewed in [8]). CR3-mediated phagocytosis can be synergistically enhanced by other receptors, such as CR1, CD14, and scavenger receptors, in the internalization of pathogens like *Francisella tularensis* [77], and *Borrelia burgdorferi* [78]. Considering that CD13 also acts as a co-receptor to other phagocytic receptors like FcγRs and mannose receptors [35,49], it is plausible that a similar functional interaction exists between CD13 and CR3, as it does between CD44 and CR3, where CD44-mediated phagocytosis triggers, and is partially dependent on, CD11b activation [79].

CD13 is overexpressed in many cancers, whereby adhesion and cell motility, a mechanistically closely related phenomenon, contribute decisively to tumor progression [80,81,82]. The peptidase activity of CD13 has long been implicated in the ability of myeloid leukemia cells to resist apoptosis. Professor Kiyohiko Hatake’s research group at the Japanese Foundation of Cancer Research has dedicated decades to investigating this phenomenon and has reported that when leukemic cells attach to vascular endothelial cells, CD13 facilitates the degradation of the pro-apoptotic cytokine IL-8 produced by the endothelium [83,84]. Building on the findings presented in this study, we propose that CD13 plays a dual role in this process. Initially, it promotes the attachment of leukemic cells to the endothelium—a critical step in any metastatic cascade—by activating CR3 and potentially other adhesion molecules. Subsequently, its peptidase activity aids in cell survival by breaking down pro-apoptotic molecules secreted by the vasculature.

Therefore, future research should focus on understanding the functional impact of CD13 crosslinking on CR3-mediated adhesion and phagocytosis and identifying the specific functions that are coordinated by these receptors. It will also be necessary to assess the participation of other components of the proposed signaling pathway governing CD13-mediated CR3 activation and membrane expression both in vitro and, eventually, in vivo. One possible approach is to use a CRISPR-Cas9 screening strategy, disrupting the genes encoding the signaling pathway components individually in immortalized cells and subsequently expanding them into cell lines. This would enable the characterization of various aspects of the signaling pathway, including the timing of events and the consequences of the absence of each protein. Additionally, the phosphorylation at serine 8 and 10 in the cytoplasmic tail of CD13, which has not been reported yet, should be evaluated as it could add extra docking sites for accessory proteins. Such specifics could provide the basis for the design of therapies that inhibit or enhance particular cellular activities to prevent the spread of cancers in which CD13 is overexpressed.

## 5. Conclusions

In conclusion, the understanding of CD13 has evolved from being a leukemia marker to a co-receptor and a moonlighting enzyme. This study reveals that CD13 not only elicits outside-in signaling but also triggers inside-out signaling, leading to the activation and membrane expression of CR3 (CD11b/CD18), another immune receptor. These findings highlight the ability of CD13 to induce cell phenomena comparable to classical phagocytic receptors, despite the absence of canonical signaling motifs.

## Data Availability

The data presented in this study are available within this article and its supplementary material.

## Supplementary Materials

The following supporting information can be downloaded at: https://www.mdpi.com/article/10.3390/biom13101488/s1, Figure S1: Workflow; Figure S2: Rise in CD11b expression after differentiation of THP-1 monocytes into macrophages; Figure S3: CD13 crosslinking activates CR3 in human MDMs; Figure S4: The inhibition of Syk augments the activation of CR3 (CD11b/CD18) triggered by CD13 crosslinking on MDMs; Figure S5: The proportion of activated CD11b relative to its total membrane expression changes with PLCγ and Src inhibitors, but not with Syk or actin polymerization ones; Figure S6: Molecular ontology within the protein interaction network; Figure S7: 65 non-redundant proteins were deemed to be of interest after the interrogation of databases using CD13, Syk, and CR3 (CD11b/CD18) as queries; Figure S8: CD13 to CR3 inside-out signaling pathway; Table S1: Description of the 76 proteins contained in the CD13, CR3 (CD11b/CD18), and Syk interaction network [3,85,86,87,88,89,90,91,92,93,94,95,96,97,98,99,100,101,102,103,104,105].

## References

[1] Riemann D., Kehlen A., Langner J. (1999). CD13—Not just a marker in leukemia typing. Immunol. Today.

[2] Wong A.H.M., Zhou D., Rini J.M. (2012). The X-ray crystal structure of human aminopeptidase N reveals a novel dimer and the basis for peptide processing. J. Biol. Chem..

[3] Licona-Limón I., Garay-Canales C.A., Muñoz-Paleta O., Ortega E. (2015). CD13 mediates phagocytosis in human monocytic cells. J. Leukoc. Biol..

[4] Garay-Canales C.A., Licona-Limón I., Ortega E. (2018). Distinct Epitopes on CD13 Mediate Opposite Consequences for Cell Adhesion. BioMed Res. Int..

[5] Subramani J., Ghosh M., Rahman M.M., Caromile L.A., Gerber C., Rezaul K., Han D.K., Shapiro L.H. (2013). Tyrosine Phosphorylation of CD13 Regulates Inflammatory Cell–Cell Adhesion and Monocyte Trafficking. J. Immunol..

[6] Bednarczyk M., Stege H., Grabbe S., Bros M. (2020). β2 Integrins—Multi-Functional Leukocyte Receptors in Health and Disease. Int. J. Mol. Sci..

[7] Bouti P., Webbers S.D.S., Fagerholm S.C., Alon R., Moser M., Matlung H.L., Kuijpers T.W. (2021). β2 Integrin Signaling Cascade in Neutrophils: More Than a Single Function. Front. Immunol..

[8] Erdei A., Lukácsi S., Mácsik-Valent B., Nagy-Baló Z., Kurucz I., Bajtay Z. (2019). Non-identical twins: Different faces of CR3 and CR4 in myeloid and lymphoid cells of mice and men. Semin. Cell Dev. Biol..

[9] Sung S. (2020). Co-immunostaining of ICAM-1, ICAM-2, and CD31 in Mouse Kidney Glomeruli. Bio-Protocol.

[10] Wen L., Marki A., Roy P., McArdle S., Sun H., Fan Z., Gingras A.R., Ginsberg M.H., Ley K. (2021). Kindlin-3 recruitment to the plasma membrane precedes high-affinity b2-integrin and neutrophil arrest from rolling. Blood.

[11] Torres-Gomez A., Sanchez-Trincado J.L., Toribio V., Torres-Ruiz R., Rodríguez-Perales S., Yáñez-Mó M., Reche P.A., Cabañas C., Lafuente E.M. (2020). RIAM-VASP Module Relays Integrin Complement Receptors in Outside-In Signaling Driving Particle Engulfment. Cells.

[12] Wang D., Guo Q., Wei A., Li A. (2018). Differential Binding of Active and Inactive Integrin to Talin. Protein J..

[13] Retta S.F., Balzac F., Avolio M. (2006). Rap1: A turnabout for the crosstalk between cadherins and integrins. Eur. J. Cell Biol..

[14] Xue L., Geahlen R.L., Tao W.A. (2013). Identification of direct tyrosine kinase substrates based on protein kinase assay-linked phosphoproteomics. Mol. Cell. Proteom..

[15] Orsó E., Werner T., Wolf Z., Bandulik S., Kramer W., Schmitz G. (2006). Ezetimib influences the expression of raft-associated antigens in human monocytes. Cytom. Part A.

[16] Martinez V.J., Asico L.D., Jose P.A., Tiu A.C. (2020). Lipid rafts and dopamine receptor signaling. Int. J. Mol. Sci..

[17] Lingwood D., Simons K. (2010). Lipid rafts as a membrane-organizing principle. Science.

[18] Petty H.R., Worth R.G., Todd R.F. (2002). Interactions of integrins with their partner proteins in leukocyte membranes. Immunol. Res..

[19] Jongstra-Bilen J., Harrison R., Grinstein S. (2003). Fcγ-receptors Induce Mac-1 (CD11b/CD18) Mobilization and Accumulation in the Phagocytic Cup for Optimal Phagocytosis. J. Biol. Chem..

[20] Carrascal M.A., Silva M., Ferreira J.A., Azevedo R., Ferreira D., Silva A.M.N., Ligeiro D., Santos L.L., Sackstein R., Videira P.A. (2018). A functional glycoproteomics approach identifies CD13 as a novel E-selectin ligand in breast cancer. Biochim. Biophys. Acta (BBA)-Gen. Subj..

[21] Ghosh M., Lo R., Ivic I., Aguilera B., Qendro V., Devarakonda C., Shapiro L.H. (2019). CD13 tethers the IQGAP1-ARF6-EFA6 complex to the plasma membrane to promote ARF6 activation, B1 integrin recycling, and cell migration. Sci. Signal..

[22] Shooshtari P., Fortuno E.S., Blimkie D., Yu M., Gupta A., Kollmann T.R., Brinkman R.R. (2010). Correlation analysis of intracellular and secreted cytokines via the generalized integrated mean fluorescence intensity. Cytom. Part A.

[23] Szklarczyk D., Gable A.L., Lyon D., Junge A., Wyder S., Huerta-Cepas J., Simonovic M., Doncheva N.T., Morris J.H., Bork P. (2019). STRING v11: Protein-protein association networks with increased coverage, supporting functional discovery in genome-wide experimental datasets. Nucleic Acids Res..

[24] von Mering C., Jensen L.J., Snel B., Hooper S.D., Krupp M., Foglierini M., Jouffre N., Huynen M.A., Bork P. (2005). STRING: Known and predicted protein-protein associations, integrated and transferred across organisms. Nucleic Acids Res..

[25] Stelzer G., Rosen N., Plaschkes I., Zimmerman S., Twik M., Fishilevich S., Stein T.I., Nudel R., Lieder I., Mazor Y. (2016). The GeneCards suite: From gene data mining to disease genome sequence analyses. Curr. Protoc. Bioinform..

[26] NCBI Resource Coordinators (2018). Database resources of the National Center for Biotechnology Information. Nucleic Acids Res..

[27] Santos A.N., Langner J., Herrmann M., Riemann D. (2000). Aminopeptidase N/CD13 is directly linked to signal transduction pathways in monocytes. Cell. Immunol..

[28] Mina-Osorio P., Winnicka B., O’Conor C., Grant C.L., Vogel L.K., Rodriguez-Pinto D., Holmes K.V., Ortega E., Shapiro L.H. (2008). CD13 is a novel mediator of monocytic/endothelial cell adhesion. J. Leukoc. Biol..

[29] López-Cortés G.I., Díaz-Alvarez L., Ortega E. (2021). Leukocyte Membrane Enzymes Play the Cell Adhesion Game. Front. Immunol..

[30] Mazumder A., Shiao S., Haricharan S. (2021). HER2 Activation and Endocrine Treatment Resistance in HER2-negative Breast Cancer. Endocrinology.

[31] Hynes R.O. (2002). Integrins: Bidirectional, allosteric signaling machines. Cell.

[32] Lefort C.T., Hyun Y.-M., Schultz J.B., Law F.-Y., Waugh R.E., Knauf P.A., Kim M. (2009). Outside-In Signal Transmission by Conformational Changes in Integrin Mac-1. J. Immunol..

[33] Harokopakis E., Hajishengallis G. (2005). Integrin activation by bacterial fimbriae through a pathway involving CD14, Toll-like receptor 2, and phosphatidylinositol-3-kinase. Eur. J. Immunol..

[34] Han C., Jin J., Xu S., Liu H., Li N., Cao X. (2010). Integrin CD11b negatively regulates TLR-triggered inflammatory responses by activating Syk and promoting degradation of MyD88 and TRIF via Cbl-b. Nat. Immunol..

[35] Mina-Osorio P., Ortega E. (2005). Aminopeptidase N (CD13) functionally interacts with FcγRs in human monocytes. J. Leukoc. Biol..

[36] Pérez-Figueroa E., Álvarez-Carrasco P., Ortega E. (2022). Crosslinking of membrane CD13 in human neutrophils mediates phagocytosis and production of reactive oxygen species, neutrophil extracellular traps and proinflammatory cytokines. Front. Immunol..

[37] Martínez de Paz A., Zvi Josefowicz S., Alexia Martínez de Paz C. (2021). Signaling-to-chromatin pathways in the immune system. Immunol. Rev..

[38] Nuutila J., Jalava-Karvinen P., Hohenthal U., Laitinen I., Kotilainen P., Rajamäki A., Nikoskelainen J., Lilius E.M. (2009). Comparison of degranulation of easily mobilizable intracellular granules by human phagocytes in healthy subjects and patients with infectious diseases. Hum. Immunol..

[39] Jang J.-W., Park S., Moon E.-Y. (2021). Spleen tyrosine kinase regulates crosstalk of hypoxia-inducible factor-1α and nuclear factor (erythroid-derived2)-like 2 for B cell survival. Int. Immunopharmacol..

[40] Sahan-Firat S., Temiz-Resitoglu M., Guden D.S., Senol S.P., Sari A.N., Cil M., Unsal D., Korkmaz B., Tunctan B., Malik K.U. (2019). NF-κB activation mediates LPS-or zymosan-induced hypotension and inflammation reversed by BAY61-3606, a selective Syk inhibitor, in rat models of septic and non-septic shock. Clin. Exp. Pharmacol. Physiol..

[41] Zheng T.J., Lofurno E.R., Melrose A.R., Lakshmanan H.H.S., Pang J., Phillips K.G., Fallon M.E., Kohs T.C., Ngo A.T., Shatzel J.J. (2021). Assessment of the effects of Syk and BTK inhibitors on GPVI-mediated platelet signaling and function. Am. J. Physiol. Cell Physiol..

[42] Saci A., Liu W.Q., Vidal M., Garbay C., Rendu F., Bachelot-Loza C. (2002). Differential effect of the inhibition of Grb2-SH3 interactions in platelet activation induced by thrombin and by Fc receptor engagement. Biochem. J..

[43] Mina-Osorio P., Shapiro L.H., Ortega E. (2006). CD13 in cell adhesion: Aminopeptidase N (CD13) mediates homotypic aggregation of monocytic cells. J. Leukoc. Biol..

[44] Torres-Gomez A., Cabañas C., Lafuente E.M. (2020). Phagocytic Integrins: Activation and Signaling. Front. Immunol..

[45] Mendoza-Coronel E., Ortega E. (2017). Macrophage polarization modulates FcγR- and CD13-mediated phagocytosis and reactive oxygen species production, independently of receptor membrane expression. Front. Immunol..

[46] Kühlmann U.C., Chwieralski C.E., van den Brule S., Röcken C., Reinhold D., Welte T., Bühling F. (2009). Modulation of cytokine production and silica-induced lung fibrosis by inhibitors of aminopeptidase N and of dipeptidyl peptidase-IV-related proteases. Life Sci..

[47] Kehlen A., Göhring B., Langner J., Riemann D. (2001). Regulation of the expression of aminopeptidase A aminopeptidase N/CD13 and dipeptidylpeptidase IV/CD26 in renal carcinoma cells and renal tubular epithelial cells by cytokines and cAMP-increasing mediators. Clin. Exp. Immunol..

[48] Gabrilovac J., Breljak D., Čupić B. (2008). Regulation of aminopeptidase N (EC 3.4.11.2; APN; CD13) on the HL-60 cell line by TGF-β1. Int. Immunopharmacol..

[49] Gabrilovac J., Čupić B., Živković E., Horvat L., Majhen D. (2011). Expression, regulation and functional activities of aminopeptidase N (EC 3.4.11.2; APN; CD13) on murine macrophage J774 cell line. Immunobiology.

[50] Villaseñor-Cardoso M.I., Frausto-Del-Río D.A., Ortega E. (2013). Aminopeptidase N (CD13) Is involved in phagocytic processes in human dendritic cells and macrophages. BioMed Res. Int..

[51] Shabir K., Gharanei S., Orton S., Patel V., Chauhan P., Karteris E., Randeva H.S., Brown J.E., Kyrou I. (2022). Asprosin Exerts Pro-Inflammatory Effects in THP-1 Macrophages Mediated via the Toll-like Receptor 4 (TLR4) Pathway. Int. J. Mol. Sci..

[52] Souissi C., Marzouki S., Elbini-Dhouib I., Jebali J., Oliveira F., Valenzuela J.G., Srairi-Abid N., Kamhawi S., Ben Ahmed M. (2023). PpSP32, the Phlebotomus papatasi immunodominant salivary protein, exerts immunomodulatory effects on human monocytes, macrophages, and lymphocytes. Parasit. Vectors.

[53] Zhou J., Fang F., Qi J., Li T., Zhang L., Liu H., Lv J., Xu T., Wu F., Song C. (2022). Activation of Nrf2 modulates protective immunity against Mycobacterium tuberculosis infection in THP1-derived macrophages. Free Radic. Biol. Med..

[54] Lee A.J., Ashkar A.A. (2018). The dual nature of type I and type II interferons. Front. Immunol..

[55] Gredmark S., Britt W.B., Xie X., Lindbom L., Söderberg-Nauclér C. (2004). Human Cytomegalovirus Induces Inhibition of Macrophage Differentiation by Binding to Human Aminopeptidase N/CD13. J. Immunol..

[56] Yeager C., Ashmun R., Williams R., Cardellichio C., Shapiro L., Look T., Holmes K. (1992). Human aminopeptidase N is a receptor for human coronavirus 229E. Nature.

[57] Yamaya M., Nishimura H., Deng X., Sugawara M., Watanabe O., Nomura K., Shimotai Y., Momma H., Ichinose M., Kawase T. (2020). Inhibitory effects of glycopyrronium, formoterol, and budesonide on coronavirus HCoV-229E replication and cytokine production by primary cultures of human nasal and tracheal epithelial cells. Respir. Investig..

[58] Trinchieri G. (2003). Interleukin-12 and the regulation of innate resistance and adaptive immunity. Nat. Rev. Immunol..

[59] Teng M.W.L., Bowman E.P., McElwee J.J., Smyth M.J., Casanova J.L., Cooper A.M., Cua D.J. (2015). IL-12 and IL-23 cytokines: From discovery to targeted therapies for immune-mediated inflammatory diseases. Nat. Med..

[60] Vignali D.A.A., Kuchroo V.K. (2012). IL-12 family cytokines: Immunological playmakers. Nat. Immunol..

[61] Stanley A.C., Lieu Z.Z., Wall A.A., Venturato J., Khromykh T., Hamilton N.A., Gleeson P.A., Stow J.L. (2012). Recycling endosome-dependent and -independent mechanisms for IL-10 secretion in LPS-activated macrophages. J. Leukoc. Biol..

[62] Stow J.L., Murray R.Z. (2013). Intracellular trafficking and secretion of inflammatory cytokines. Cytokine Growth Factor Rev..

[63] Manderson A.P., Kay J.G., Hammond L.A., Brown D.L., Stow J.L. (2007). Subcompartments of the macrophage recycling endosome direct the differential secretion of IL-6 and TNFα. J. Cell Biol..

[64] Ge Y., Huang M., Yao Y.M. (2020). Biology of Interleukin-17 and Its Pathophysiological Significance in Sepsis. Front. Immunol..

[65] Hou Y., Zhu L., Tian H., Sun H.X., Wang R., Zhang L., Zhao Y. (2018). IL-23-induced macrophage polarization and its pathological roles in mice with imiquimod-induced psoriasis. Protein Cell.

[66] Erbel C., Dengler T.J., Wangler S., Lasitschka F., Bea F., Wambsganss N., Hakimi M., Böckler D., Katus H.A., Gleissner C.A. (2011). Expression of IL-17A in human atherosclerotic lesions is associated with increased inflammation and plaque vulnerability. Basic Res. Cardiol..

[67] Bozinovski S., Seow H.J., Chan S.P.J., Anthony D., McQualter J., Hansen M., Jenkins B.J., Anderson G.P., Vlahos R. (2015). Innate cellular sources of interleukin-17A regulate macrophage accumulation in cigarette- smoke-induced lung inflammation in mice. Clin. Sci..

[68] Miller J.E., Ahn S.H., Marks R.M., Monsanto S.P., Fazleabas A.T., Koti M., Tayade C. (2020). IL-17A Modulates Peritoneal Macrophage Recruitment and M2 Polarization in Endometriosis. Front. Immunol..

[69] Ferreira N., Mesquita I., Baltazar F., Silvestre R., Granja S. (2020). IL-17A and IL-17F orchestrate macrophages to promote lung cancer. Cell. Oncol..

[70] Gabrilovac J., Breljak D., Čupić B., Ambriović-Ristov A. (2005). Regulation of aminopeptidase N (EC 3.4.11.2; APN; CD13) by interferon-γ on the HL-60 cell line. Life Sci..

[71] Morgan R.L., Behbahani-Nejad N., Endres J., Amin M.A., Lepore N.J., Du Y., Urquhart A., Chung K.C., Fox D.A. (2016). Localization, Shedding, Regulation and Function of Aminopeptidase N/CD13 on Fibroblast like Synoviocytes. PLoS ONE.

[72] Mina-Osorio P., Soto-Cruz I., Ortega E. (2007). A role for galectin-3 in CD13-mediated homotypic aggregation of monocytes. Biochem. Biophys. Res. Commun..

[73] Fiddler C.A., Parfrey H., Cowburn A.S., Luo D., Nash G.B., Murphy G., Chilvers E.R. (2016). The aminopeptidase CD13 induces homotypic aggregation in neutrophils and impairs collagen invasion. PLoS ONE.

[74] Kido A., Krueger S., Haeckel C., Roessner A. (2003). Inhibitory effect of antisense aminopeptidase N (APN/CD13) cDNA transfection on the invasive potential of osteosarcoma cells. Clin. Exp. Metastasis.

[75] Berthold T., Glaubitz M., Muschter S., Groß S., Palankar R., Reil A., Helm C.A., Bakchoul T., Schwertz H., Bux J. (2015). Human neutrophil antigen-3a antibodies induce neutrophil stiffening and conformational activation of CD11b without shedding of L-selectin. Transfusion.

[76] Li N., Yang H., Wang M., Lü S., Zhang Y., Long M. (2018). Ligand-specific binding forces of LFA-1 and Mac-1 in neutrophil adhesion and crawling. Mol. Biol. Cell.

[77] Schwartz J.T., Barker J.H., Long M.E., Kaufman J., McCracken J., Allen L.-A.H. (2012). Natural IgM Mediates Complement-Dependent Uptake of Francisella tularensis by Human Neutrophils via Complement Receptors 1 and 3 in Nonimmune Serum. J. Immunol..

[78] Gonzalez A.C., Barriales D., Palacios A., Montesinos-Robledo M., Navasa N., Azkargorta M., Peña-Cearra A., Tomás-Cortázar J., Escobes I., Pascual-Itoiz M.A. (2019). Regulation of macrophage activity by surface receptors contained within Borrelia burgdorferi-enriched phagosomal fractions. PLoS Pathog..

[79] Vachon E., Martin R., Kwok V., Cherepanov V., Chow C.W., Doerschuk C.M., Plumb J., Grinstein S., Downey G.P. (2007). CD44-mediated phagocytosis induces inside-out activation of complement receptor-3 in murine macrophages. Blood.

[80] Tokuhara T., Hattori N., Ishida H., Hirai T., Higashiyama M., Kodama K., Miyake M. (2006). Clinical Significance of Aminopeptidase N in Non–Small Cell Lung Cancer. Clin. Cancer Res..

[81] Piedfer M., Dauzonne D., Tang R., N’Guyen J., Billard C., Bauvois B. (2011). Aminopeptidase-N/CD13 is a potential proapoptotic target in human myeloid tumor cells. FASEB J..

[82] Hashida H., Takabayashi A., Kanai M., Adachi M., Kondo K., Kohno N., Yamaoka Y., Miyake M. (2002). Aminopeptidase N is involved in cell motility and angiogenesis: Its clinical significance in human colon cancer. Gastroenterology.

[83] Mishima Y., Terui Y., Sugimura N., Matsumoto-Mishima Y., Rokudai A., Kuniyoshi R., Hatake K. (2007). Continuous treatment of bestatin induces anti-angiogenic property in endothelial cells. Cancer Sci..

[84] Mishima Y., Matsumoto-Mishima Y., Terui Y., Katsuyama M., Yamada M., Mori M., Ishizaka Y., Ikeda K., Watanabe J.I., Mizunuma N. (2002). Leukemic Cell-Surface CD13/Aminopeptidase N and Resistance to Apoptosis Mediated by Endothelial Cells. J. Natl. Cancer Inst..

[85] Mina-Osorio P. (2008). The moonlighting enzyme CD13: Old and new functions to target. Trends Mol. Med..

[86] Fernandes R.A., Su L., Nishiga Y., Ren J., Bhuiyan A.M., Cheng N., Kuo C.J., Picton L.K., Ohtsuki S., Majzner R.G. (2020). Immune receptor inhibition through enforced phosphatase recruitment. Nature.

[87] Zeke A., Misheva M., Reményi A., Bogoyevitch M.A. (2016). JNK Signaling: Regulation and Functions Based on Complex Protein-Protein Partnerships. Microbiol. Mol. Biol. Rev..

[88] Meier T.I., Cook J.A., Thomas J.E., Radding J.A., Horn C., Lingaraj T., Smith M.C. (2004). Cloning, expression, purification, and characterization of the human Class Ia phosphoinositide 3-kinase isoforms. Protein Expr. Purif..

[89] Guyot B., Mouchiroud G. (2003). Characterization of promoter elements directing Mona/Gads molecular adapter expression in T and myelomonocytic cells: Involvement of the AML-1 transcription factor. J. Leukoc. Biol..

[90] Oda A., Ochs H.D., Lasky L.A., Spencer S., Ozaki K., Fujihara M., Handa M., Ikebuchi K., Ikeda H. (2001). CrkL is an adapter for Wiskott-Aldrich syndrome protein and Syk. Blood.

[91] Shi X., Pan S., Li Y., Ma W., Wang H., Xu C., Li L. (2020). Xanthoplanine attenuates macrophage polarization towards M1 and inflammation response via disruption of CrkL-STAT5 complex. Arch. Biochem. Biophys..

[92] Platanias L.C., Uddin S., Bruno E., Korkmaz M., Ahmad S., Alsayed Y., van den Berg D., Druker B.J., Wickrema A., Hoffman R. (1999). CrkL and CrkII participate in the generation of the growth inhibitory effects of interferons on primary hematopoietic progenitors. Exp. Hematol..

[93] Kataoka T.R., Kumanogoh A., Bandara G., Metcalfe D.D., Gilfillan A.M. (2010). CD72 Negatively Regulates KIT-Mediated Responses in Human Mast Cells. J. Immunol..

[94] Cui Y., Zhou F., Bai H., Wei L., Tan J., Zeng Z., Song Q., Chen J., Huang N. (2018). Real-time QCM-D monitoring of endothelial cells and macrophages adhering and spreading to SEMA4D/heparin surfaces. Colloids Surf. B Biointerfaces.

[95] Galuppo M.K., de Rezende E., Forti F.L., Cortez M., Cruz M.C., Teixeira A.A., Giordano R.J., Stolf B.S. (2018). CD100/Sema4D increases macrophage infection by Leishmania (Leishmania) amazonensis in a CD72 dependent manner. Front. Microbiol..

[96] Irizarry-Caro R.A., McDaniel M.M., Overcast G.R., Jain V.G., Troutman T.D., Pasare C. (2020). TLR signaling adapter BCAP regulates inflammatory to reparatory macrophage transition by promoting histone lactylation. Proc. Natl. Acad. Sci. USA.

[97] Miao Y., Jiang M., Qi L., Yang D., Xiao W., Fang F. (2020). BCAP Regulates Dendritic Cell Maturation Through the Dual-Regulation of NF-κB and PI3K/AKT Signaling During Infection. Front. Immunol..

[98] Troutman T.D., Hu W., Fulenchek S., Yamazaki T., Kurosaki T., Bazand J.F., Pasare C. (2012). Role for B-cell adapter for PI3K (BCAP) as a signaling adapter linking Toll-like receptors (TLRs) to serine/threonine kinases PI3K/Akt. Proc. Natl. Acad. Sci. USA.

[99] Lőrincz Á.M., Szeifert V., Bartos B., Szombath D., Mócsai A., Ligeti E. (2019). Different Calcium and Src Family Kinase Signaling in Mac-1 Dependent Phagocytosis and Extracellular Vesicle Generation. Front. Immunol..

[100] Teitelbaum S.L. (2011). The osteoclast and its unique cytoskeleton. Ann. N. Y. Acad. Sci..

[101] Hedl M., Sun R., Huang C., Abraham C. (2019). STAT3 and STAT5 Signaling Thresholds Determine Distinct Regulation for Innate Receptor–Induced Inflammatory Cytokines, and STAT3 / STAT5 Disease Variants Modulate These Outcomes. J. Immunol..

[102] Hyduk S.J., Rullo J., Cano A.P., Xiao H., Chen M., Moser M., Cybulsky M.I. (2011). Talin-1 and Kindlin-3 Regulate α 4 β 1 Integrin-Mediated Adhesion Stabilization, but Not G Protein-Coupled Receptor-Induced Affinity Upregulation. J. Immunol..

[103] Kim M., Carman C.V., Springer T.A. (2003). Bidirectional transmembrane signaling by cytoplasmic domain separation in integrins. Science.

[104] Lim J., Thompson J., May R.C., Hotchin N.A., Caron E. (2013). Regulator of G-Protein Signalling-14 (RGS14) Regulates the Activation of αMβ2 Integrin during Phagocytosis. PLoS ONE.

[105] Senetar M.A., McCann R.O. (2005). Gene duplication and functional divergence during evolution of the cytoskeletal linker protein talin. Gene.

