# Supplementary material for: Aminopeptidase N/CD13 Crosslinking Promotes the Activation and Membrane Expression of Integrin CD11b/CD18
Source: Biomolecules. 2023 Oct 6;13(10):1488. doi: 10.3390/biom13101488 (PMC10604325; doi:10.3390/biom13101488)
Supplement: Supplementary file 1 [file biomolecules-13-01488-s001.zip › Supplementary Figure.pdf]

Supplementary Material

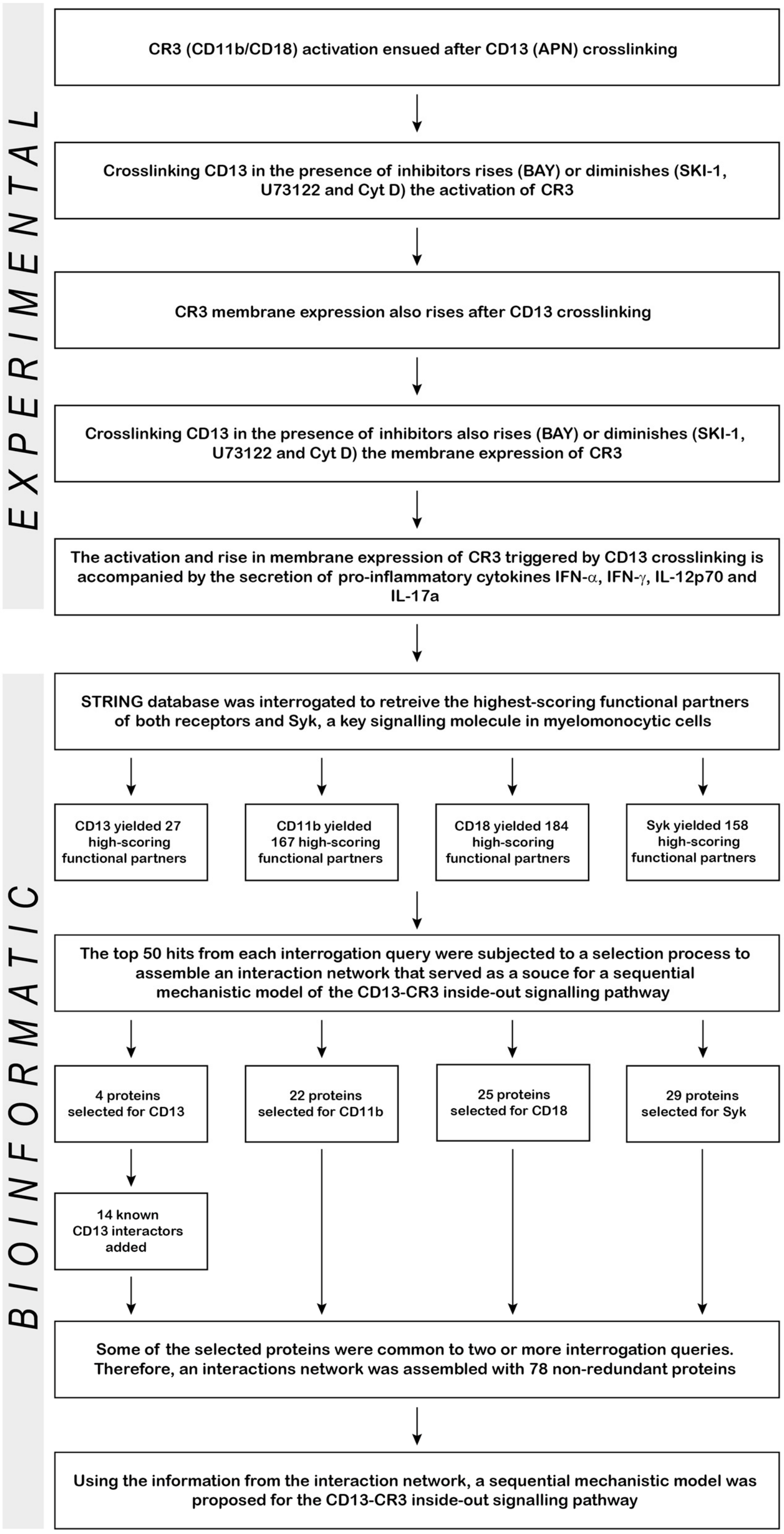

Supplementary Figure S1. Workflow. Flow diagram briefly describing each step taken to tackle the task of proposing a sequential mechanistic model for the newly described CD13-CR3 inside-out signaling pathway.

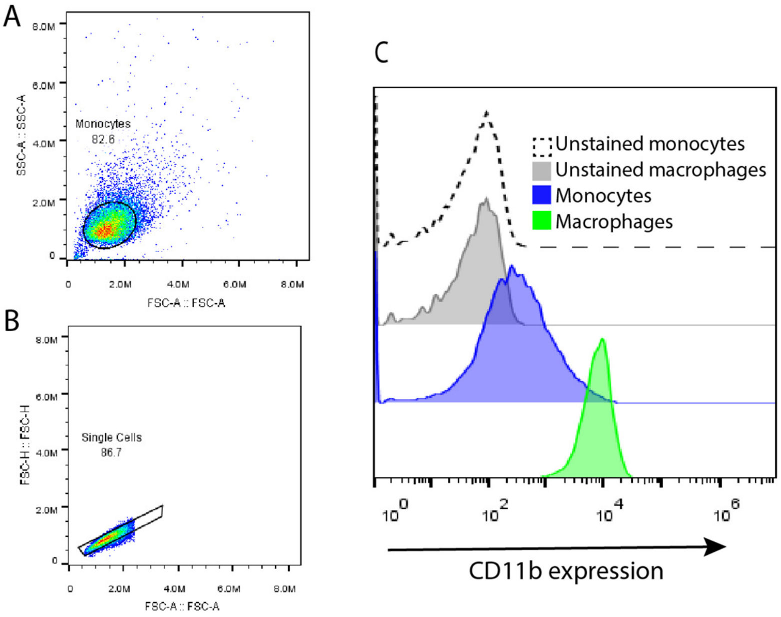

Supplementary Figure S2. Rise in CD11b expression after differentiation of THP-1 monocytes into macrophages (A) Cells were first gated for size and granularity, then for **(B)** singlets, and finally for **(C)** median fluorescence intensity in the RL1 (APC) channel. **(C)** Representative histograms from THP-1 cells before (monocytes) and after differentiation (macrophages) with PMA.

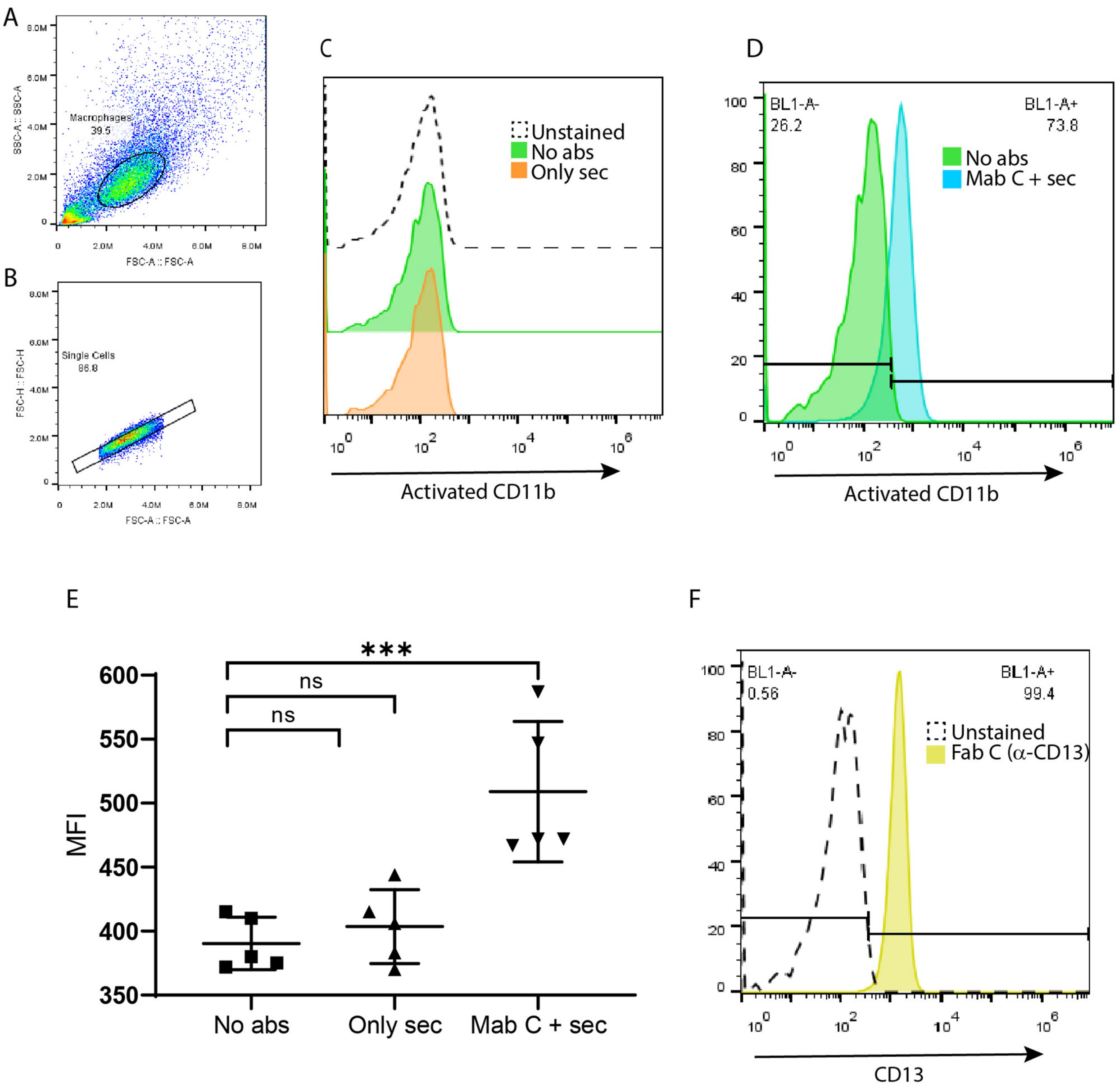

Supplementary Figure S3. CD13 crosslinking activates CR3 in human MDMs (A) Cells first gated for size and granularity, then for **(B)** singlets, and finally for **(C and D)** median fluorescence intensity in the BL1 (FITC) channel. **(C)** Controls. **(D)** Representative histograms from a sample crosslinked with C (anti-CD13) and secondary antibodies vs its control without antibodies. **(E)** Average and standard deviations from 5 independent experiments. ***<0.001, ns= non-significant. **(F)** Representative histogram demonstrating that virtually all cells are positive for the CD13 stain.

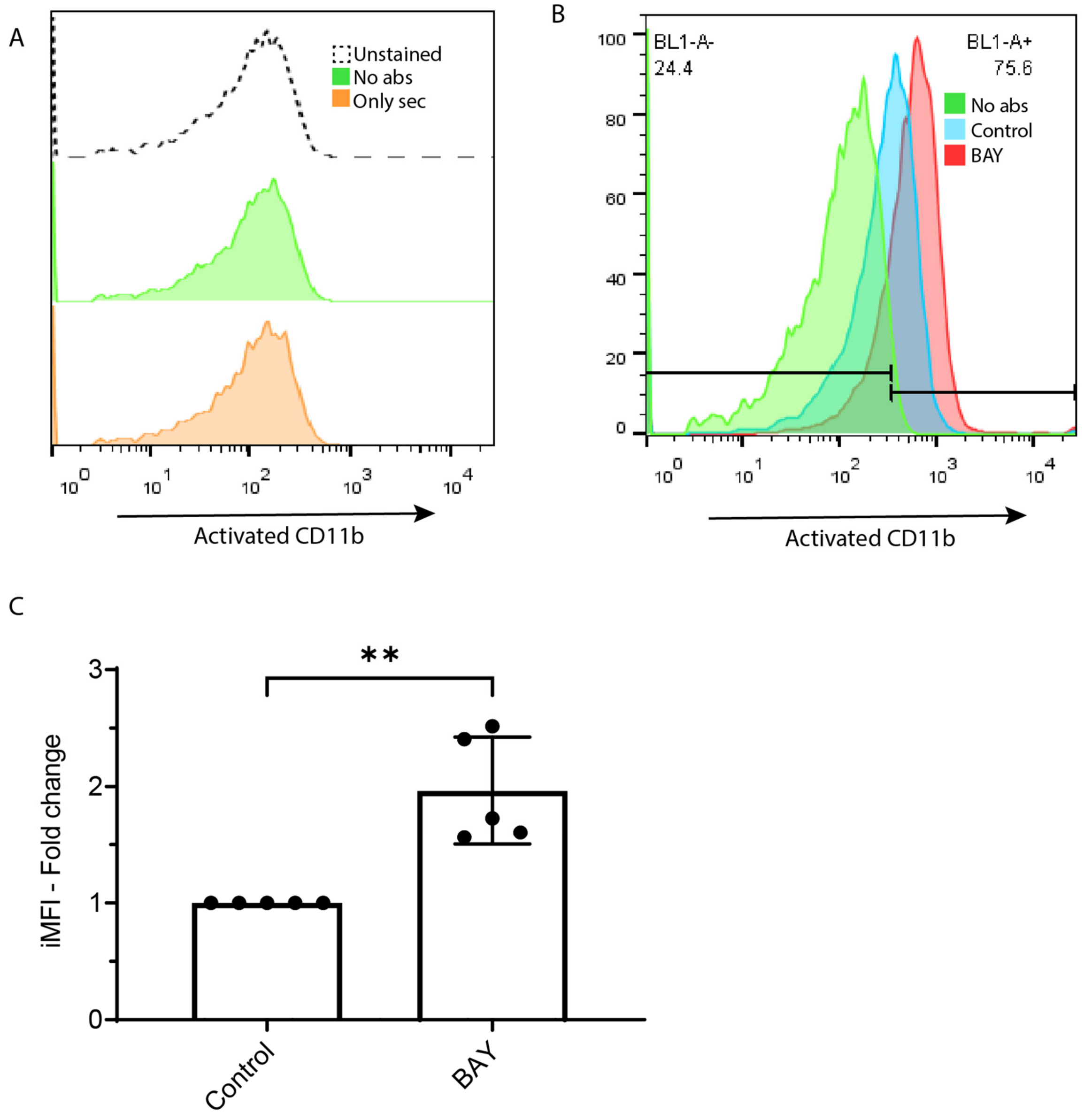

Supplementary Figure S4. The inhibition of Syk augments the activation of CR3 (CD11b/CD18) triggered by CD13 crosslinking on MDMs. (A) Representative histograms from unstained cells and, cells treated without crosslinking antibodies or only with secondary antibody stained with anti-CD11b(activated)-FITC antibody. **(B)** Average and standard deviations of the iMFI from 5 independent experiments. **<0.01.

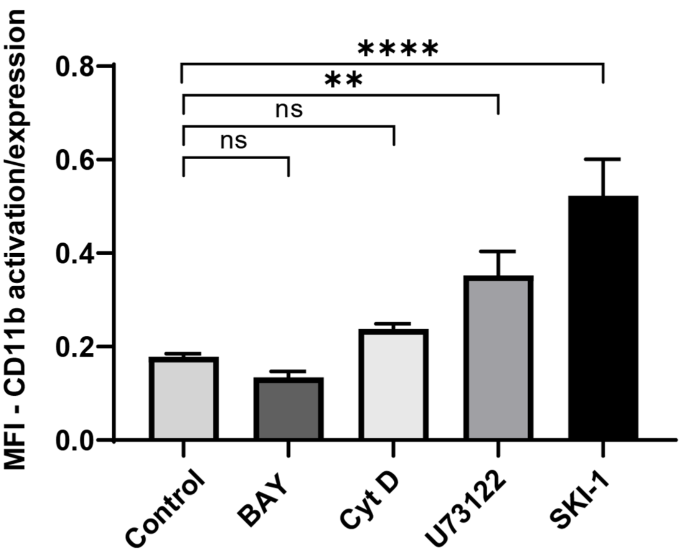

Supplementary Figure S5. The proportion of activated CD11b relative to its total membrane expression changes with PLCγ and Src inhibitors, but not with Syk or actin polymerization ones. Average and standard deviations of the relation of CD11b activation/expression in cells with crosslinked CD13 in the presence of different inhibitors, n=3, **<0.01, ****<0.0001, ns= non-significant.

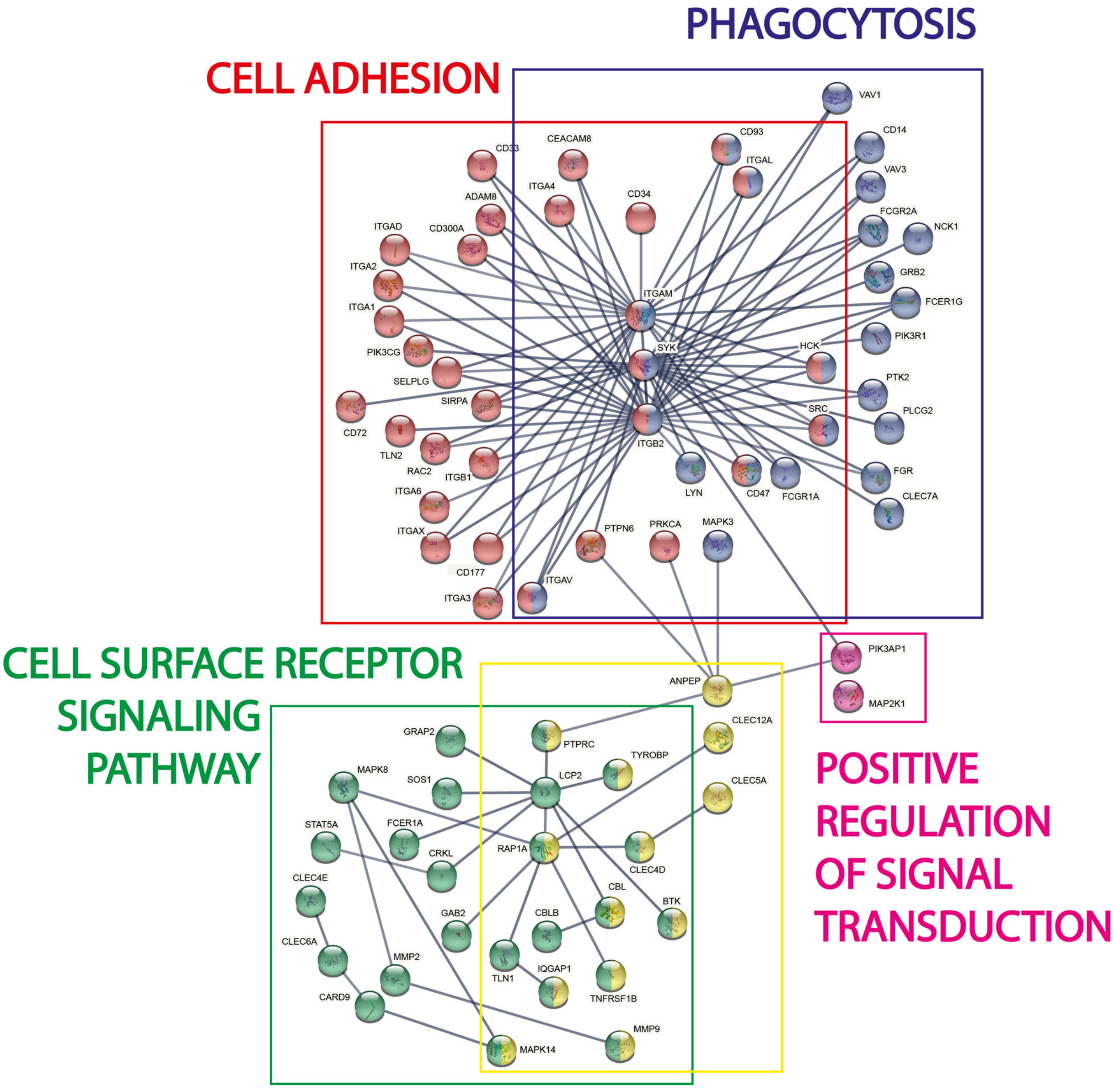

MYELOID CELL ACTIVATION INVOLVED IN IMMUNE RESPONSE

Supplementary Figure S6. Molecular ontology within the protein interaction network. Depiction of the essential functions for which these molecules were included in our interactions network. Each box encloses all the proteins within a category.

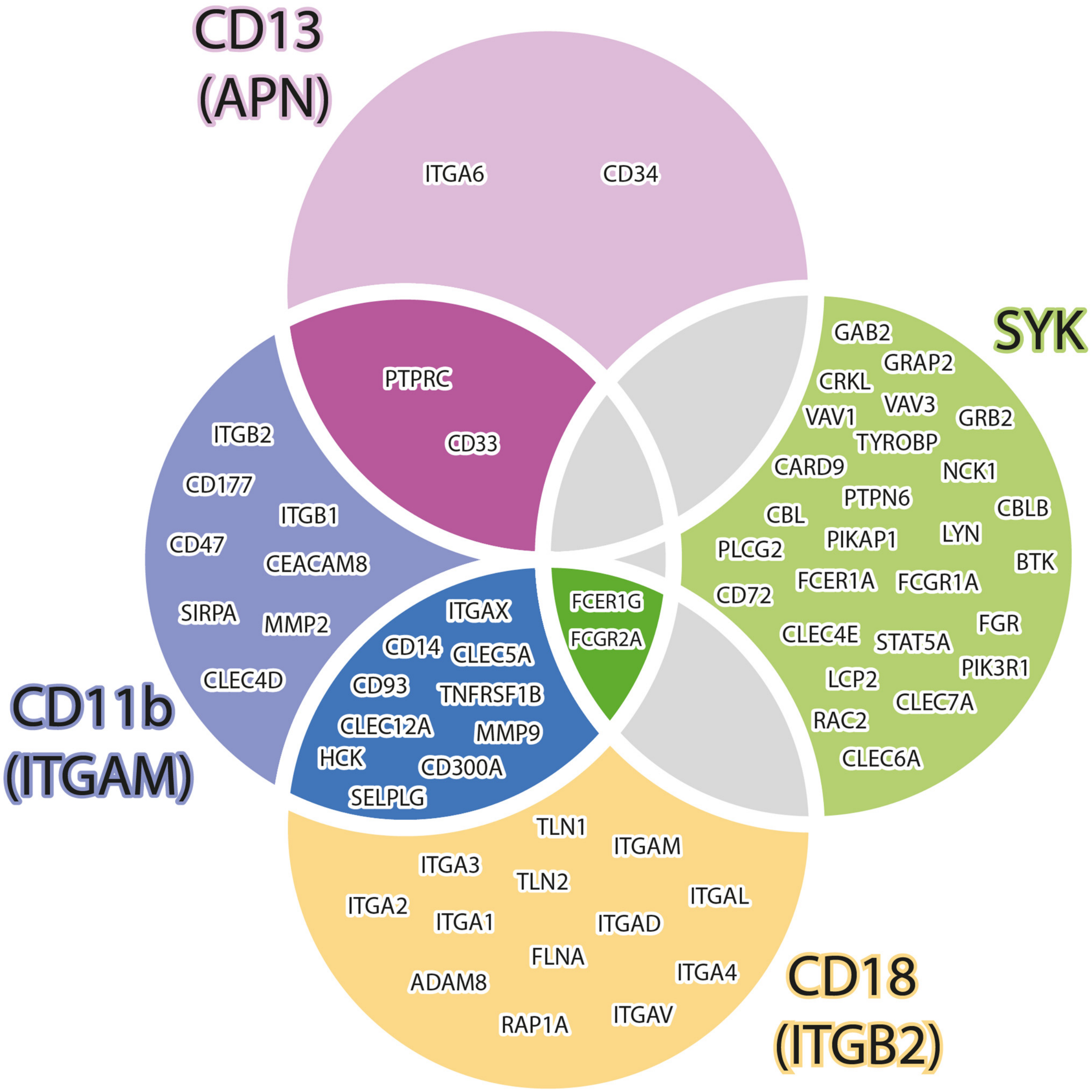

Supplementary Figure S7. 65 non-redundant proteins were deemed of interest after the interrogation of databases using CD13, Syk, and CR3 (CD11b/CD18) as queries. This Venn diagram shows each interrogation query as a circle, inside of them are the proteins of interest selected according to our criteria. The molecules that appeared in more than one interrogation/narrowing down process are inside the intersections. The grey areas represent intersections with no common proteins.

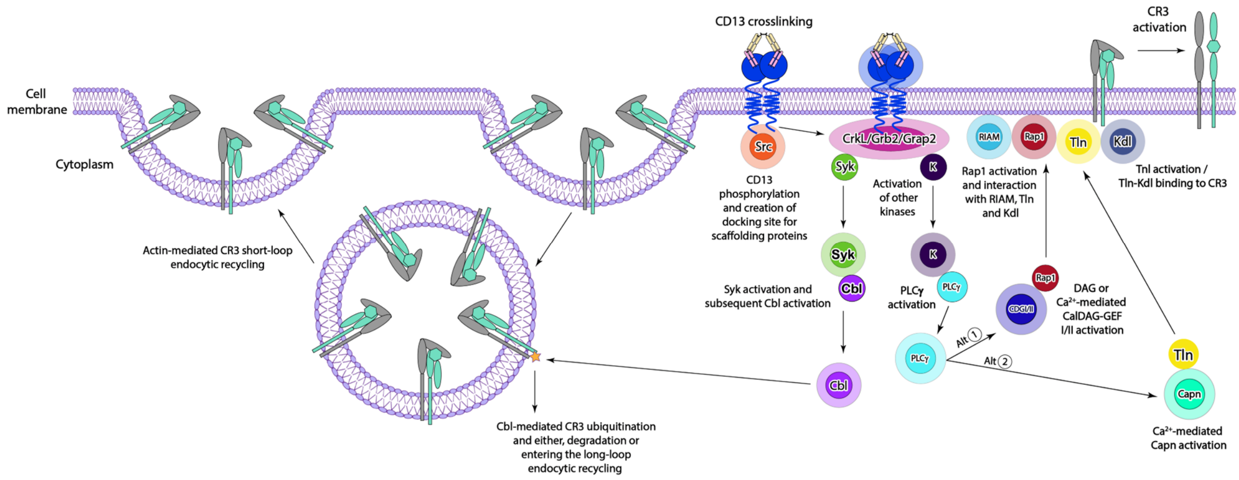

Supplementary Figure S8. CD13 to CR3 inside-out signaling pathway. Schematic representation of the proposed main events in the signaling cascade that links CD13 crosslinking to CR3 activation and membrane expression. We propose that upon antibody crosslinking, CD13 dimers are phosphorylated on their intracellular portion (Tyr6), most likely by Src [5]. This may create a docking site for scaffolding or adaptor proteins like Grb2, Grap2, or Gab2. Subsequently, other molecules like Sos1, Syk, and other non-receptor tyrosine-kinases and phosphatases such as SHP-1 (PTPN6) could be recruited and activated. Next, PLCγ would be activated by one of those kinases, for example, PI3K. PLCγ produces IP3 and DAG. Since IP3 induces Ca^{2+} release from the endoplasmic reticulum, Ca^{2+}-dependent enzymes could lead to CR3 transitioning from the low-affinity to the high-affinity state. For example, Calpain can cleave and activate Talin (TLN1/2) (reviewed in [74]) which, along with Kindlin, destabilizes the CD18-CD11b interaction by interfering with the salt bridge between the integrin subunits [75], thus activating it. Of note, Ca^{2+} release is a known effect of CD13 crosslinking by specific antibodies [4]. Another possibility is the intervention of the Rap1 GEFs CalDAG-GEFI or II, which can be activated either by the DAG directly produced by PLCγ or by the Ca^{2+} released into the cytoplasm in response to IP3 [76]. In this case, the Rap GEF would catalyze the GDP/GTP exchange in Rap1 and, after phosphorylation by a kinase-like Fak or Src, the protein RIAM would bind to Rap1 through its Ras association domain, and to PIP2 within the cell membrane via its Pleckstrin domain [77]. Rap1 also anchors to the membrane and interacts with Talin and Kindlin which, as aforementioned, can activate CR3. It is worth mentioning that all PLCs can also anchor to the plasma membrane via a Pleckstrin domain [78]. In parallel, a mechanism for controlling CR3 membrane expression may ensue. One such mechanism is a cycle of clathrin-mediated endocytosis, sorting-endosome transport, and, either membrane recycling or ubiquitination and degradation of the integrin [79]. The role of Syk in this cycle is the phosphorylation of members of the ubiquitin-protein ligase family Cbl, like Cbl and Cbl-b [80], both present in our interaction network. Therefore, these ligases may ubiquitinate a proportion of the CR3 molecules in the sorting endosome, targeting them to the late endosomal compartment, while another proportion of the endocytosed CR3 molecules may be recycled back to the membrane in a short loop partially mediated by F-actin, the target of Cyt D [81]. This is conceivable since Cbl-deficient murine bone marrow-derived mononuclear phagocytes display enhanced β2 integrin-mediated adhesion during inside-out dependent activation [82]. Of note, ubiquitination does not necessarily mean the molecules will be degraded as this chemical modification can be removed in

late endosomes and the receptors recycled back to the cytoplasm or cell membranes (long loop), as it occurs with a proportion of EGFR after signaling termination (reviewed in [83]).
